# ﻿Moth flies (Diptera, Psychodidae) of Estonia

**DOI:** 10.3897/zookeys.1221.119716

**Published:** 2024-12-10

**Authors:** Jozef Oboňa, Olavi Kurina, Ilmar Süda, Jan Ježek

**Affiliations:** 1 Department of Ecology, Faculty of Humanities and Natural Sciences, University of Prešov, 17. novembra 1, SK – 081 16 Prešov, Slovakia University of Prešov Prešov Slovakia; 2 Institute of Agricultural and Environmental Sciences, Estonian University of Life Sciences, Kreutzwaldi st 5-D, 51006 Tartu, Estonia Estonian University of Life Sciences Tartu Estonia; 3 Ilmar Süda FIE, Rõõmu tee 12-5, 50705 Tartu, Estonia Ilmar Süda FIE Tartu Estonia; 4 Department of Entomology, National Museum, Cirkusová 1740, CZ – 193 00, Praha 9 – Horní Počernice, Czech Republic National Museum Praha Czech Republic

**Keywords:** Baltic countries, biodiversity, checklist, new records, Palaearctic Region, Psychodinae, Sycoracinae, Trichomyiinae

## Abstract

A fundamental prerequisite for understanding and protecting biodiversity is the construction of a high-quality faunal database. The primary objective of this study was to address knowledge gaps in the biodiversity of the family Psychodidae in Estonia. Faunistic data on 45 species of moth flies (Diptera: Psychodidae) from Estonia are presented, including 30 new country-records. Sixteen species are considered important for nature conservation. An updated checklist of the family, comprising 71 species, is provided for the Baltic countries. Habitus photographs of selected Estonian species are also included.

## ﻿Introduction

The moth flies (Diptera: Psychodidae) are relatively well-represented in the Palaearctic Region, with nearly 800 known species (e.g., Wagner 1990, 2018; Evenhuis et al. 2007; Salmela et al. 2014). Although, the Baltic countries belong to an area where the biodiversity of the family Psychodidae has only been superficially studied. According to Pakalniškis et al. (2006), 41 species are known in Lithuania, while Latvia, on the other hand, remains almost entirely unexplored, with only one species recorded (Salmela and Vartija 2007). Likely due to their small size and the absence of a local specialist, the moth flies were largely excluded from earlier faunistic studies in Estonia. Only Remm (1956) named *Psychodaphalaenoides* (Linnaeus, 1758) as occurring in Estonia. The first and, so far, the only list of species was published by Salmela and Piirainen (2005) for Viidumäe Nature Reserve in the Island of Saaremaa. They described a new species – *Lepimormiahemiboreale* Salmela & Piirainen, 2005 – and listed 14 additional species.

A large amount of moth fly material from various research projects has accumulated in the authors’ possession over recent years. The aim of this study is to provide data on newly determined material, along with earlier published data on Estonian Psychodidae. Moreover, the list of moth flies of Baltic countries is presented.

## ﻿Materials and methods

**Figure 1.**
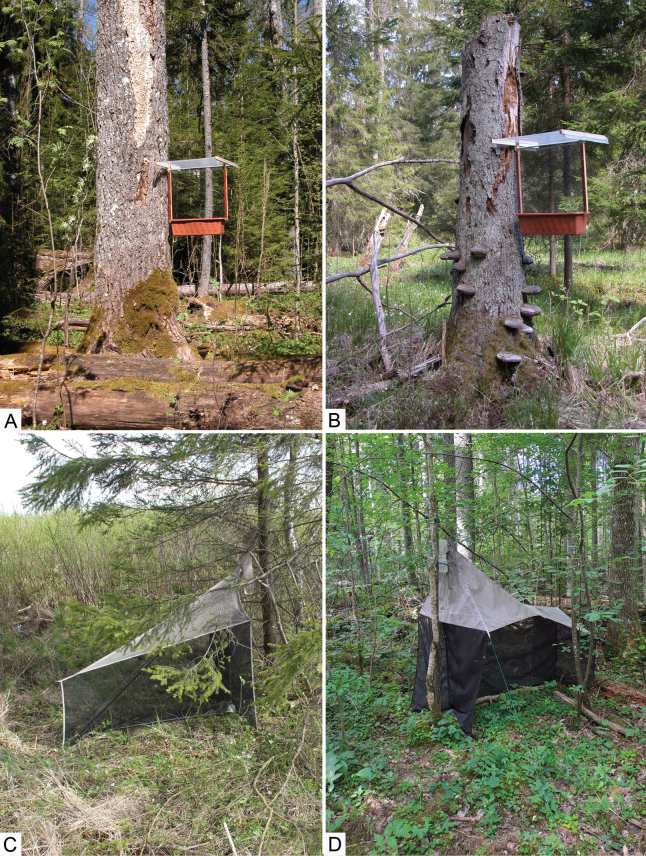
Collecting localities and the types of traps used **A** trunk window trap in Kalita NR (58°04'15"N, 24°51'37"E) **B** trunk window trap in Lahemaa NP (59°28'48"N, 25°54'24"E) **C** malaise trap near Lasila (forest patch No 75; 59°16'49.3"N, 26°13'05.2"E) **D** malaise trap in Vorbuse, Lokaatorite tee (58°25'27"N, 26°35'47"E). Photographs by IS (**A, B**), and OK (**C, D**).

**Figure 2.**
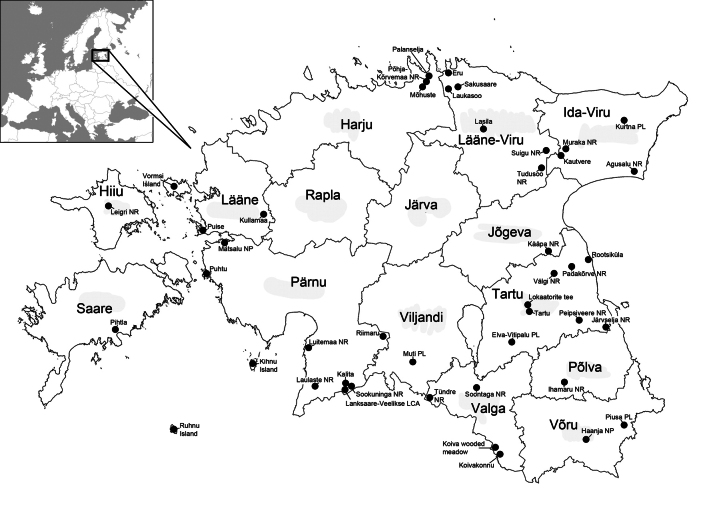
Map of Estonia showing counties (large font) and collection localities (small font). Retrieved and modified from https://geoportaal.maaamet.ee/eng/Spatial-Data/Administrative-and-Settlement-Division-p312.html (accessed 12 January 2024).

A large proportion of the material was collected from forest habitats using trunk window traps (Fig. 1A, B; abbreviated as TWT; for details see Süda (2009) and Sammet et al. (2016)). Additional material was collected using Malaise traps (Fig. 1C, D; abbreviated as MT; for details see Takkis et al. (2018) and Šikora et al. (2020)). A few specimens were collected by sweep netting or handpicking. The material was collected into ethylene or propylene glycol (in the case of TWT) or ethyl alcohol (in the case of MT, sweep netting, and handpicking). In total, material was collected from 45 localities throughout the country (Fig. 2). However, individual localities may assemble several nearby collecting spots, thus, exact geographical coordinates are provided for each sample in the studied material paragraphs.

Samples from all collection methods were sorted to order and family level prior to this study. The sorted Psychodidae specimens were stored in 70% ethyl alcohol, and the majority were identified directly under a stereomicroscope without slide-mounting. Specimens of particular interest that required detailed study were mounted on microscope slides with the following procedure: the specimens were cleared (diaphanized) using chloralphenol and subsequently treated in xylol. Cleared specimens were mounted on microscope slides using Canada balsam as the mounting media. Most of the samples were identified by the first author and were then deposited in the Insect Collection of the Institute of Agricultural and Environmental Sciences, Estonian University of Life Sciences, Tartu, Estonia (**IZBE**). Otherwise, several specimens marked with INS (= Inventory Slide Number of the family Psychodidae; see Tkoč et al. 2014) were identified by the last author. This material is deposited in the National Museum, Prague, Czech Republic (**NMPC**). Habitus photographs of selected Estonian species were compiled using LAS X software from multiple gradually focused images taken in alcohol medium by a Leica K5C camera attached to a Leica 205C stereomicroscope (see also Kjærandsen et al. 2022).

The following identification keys were used: Vaillant (1971–1983); Szabó (1983); Withers (1989a, b) and numerous unnamed original papers with descriptions of new species (e.g., Ježek 1977, 1983, 1984, 1985, 1990). The nomenclature is modified from Vaillant (1971–1983) and Wagner (1990, 2018) using the classifications of Ježek and van Harten (2005, 2009), Ježek (2007), Omelková and Ježek (2012a), Oboňa and Ježek (2014), Kvifte (2014), and Kroča and Ježek (2015, 2019, 2022). The following list of species was formatted based on similar lists from other European countries such as Ježek (2005), Oboňa and Ježek (2014), and Ježek et al. (2008, 2019, 2021b).

### ﻿List of species

In the following list of species, the examined material is organized by counties from west to east and from north to south. The paragraph of the examined material is arranged as follows: county (highlighted in bold and not repeated for each sample), sample (male or/and female, without duplicates), collecting locality, geographical coordinates, collecting time range, collecting method (in case of TWT, followed by substrate tree species), collector, and INS (if material is deposited in the National Museum, Prague).

The following abbreviations are used in the text: **LCA** – Limited conservation area, **MT** – Malaise trap, **NP** – Nature Park, **NR** – Nature Reserve, **PL** – Protected landscape, **TWT** – trunk window trap, ♂ – male, ♀ – female, **IS leg.** – Ilmar Süda leg., **OK leg.** – Olavi Kurina and Co. leg. An asterisk (*) before the species name indicates a new geographical record for Estonia.

The rarity level of each species (common, sporadically common, rare, etc.) is based on data from earlier literature, known distribution, and the abundance/frequency of the species across multiple studies. Literature sources for these assessments are provided separately for each species. Species deemed important for nature conservation are those that are considered for conservation potential/status in other European countries (e.g., Ježek and Omelková 2012; Oboňa and Ježek 2014; Kroča and Ježek 2015, 2019, 2022; Ježek et al. 2019, 2021b). In accordance with IUCN categorization (IUCN 2024), these species are assessed in Czech Republic as critically endangered (CR), endangered (EN) or nationally scarce (NS). For more information, see Omelková and Ježek (2012b).

Classification and nomenclature for individual species are provided by Ježek and van Harten (2005, 2009), Ježek (2007), Ježek et al. (2018, 2020, 2021a, b, 2023a), Omelková and Ježek (2012a), Ježek and Omelková (2012), Oboňa and Ježek (2014), Kvifte (2014), and Kroča and Ježek (2015, 2019, 2022).

## ﻿Family Psychodidae

### ﻿Subfamily Sycoracinae

#### ﻿1. Sycorax silacea

Taxon classificationAnimaliaDipteraPsychodidae

Curtis, 1839

DD14325E-C4D5-546A-8ADE-0E9C76E52704

##### Published record.

Salmela and Piirainen (2005): 304.

##### Comments.

European species, sporadically common (Ježek et al. 2020; Morelli and Biscaccianti 2021).

### ﻿Subfamily Trichomyiinae

#### ﻿*2. Trichomyia urbica

Taxon classificationAnimaliaDipteraPsychodidae

Haliday in Curtis, 1839

E78B1937-00B4-57FB-91C4-1CE76E761C7C

##### Material examined.

**Läänemaa** • ♂; Vormsi Island, Suuremõisa Park; 58°59'30"N, 23°11'34"E; 19.07.–19.08.2011; TWT (on old *Quercusrobur* L.); IS leg. **Pärnumaa** • ♂; Kihnu Island; 58°08'44"N, 23°58'14"E; 29.05.–01.07.2011; TWT (on old *Aesculushippocastanum* L.); IS leg.; • ♂; Matsalu NP; 58°42'41"N, 23°41'19"E; 11.07.–14.08.2009; TWT (on *Populustremula* L.); IS leg.; • ♂; Lanksaare-Veelikse LCA; 58°00'11"N, 24°49'42"E; 24.07.–25.08.2017; TWT (on dead *Betulapendula* Roth); IS leg. **Valgamaa** • ♂; Soontaga; 58°00'03"N, 26°05'31"E; 05.06.–06.07.2015; TWT (on *Acerplatanoides* L.); IS leg.

##### Comments.

European and Transcaucasian species (Ježek et al. 2021a, b). Important species for nature conservation, assessed as critically endangered in the Czech Republic.

### ﻿Subfamily Psychodinae

#### ﻿Mormiini

**Mormiina**

##### ﻿*3. Oomormia andrenipes

Taxon classificationAnimaliaDipteraPsychodidae

(Strobl, 1910)

E45F02C9-A778-5C57-82C5-3E22314AE309

###### Material examined.

**Lääne-Virumaa** • ♂; Tudusoo NR; 59°04'47"N, 26°45'38"E; 28.05.–25.06.2017; TWT (on *Populustremula*); IS leg.; INS 33786.

###### Comments.

Rather rare European species (Ježek 1984, 1994; Ježek and Omelková 2007; Kroča and Ježek 2022). Important species for nature conservation, assessed as critically endangered in the Czech Republic.

##### ﻿4. Lepimormia hemiboreale

Taxon classificationAnimaliaDipteraPsychodidae

Salmela & Piirainen, 2005

CA8AA621-C8F2-5176-9923-A444B2D10767

###### Published record.

Salmela and Piirainen (2005): 302.

###### Comments.

Known only from its type locality in the Viidumäe Nature Reserve, Estonia.

##### ﻿*5. Promormia eatoni

Taxon classificationAnimaliaDipteraPsychodidae

(Tonnoir, 1940)

713925D4-9A36-5806-822D-FA9008B8F566

###### Material examined.

**Pärnumaa** • ♂; Kalita NR; 58°04'15"N, 24°51'37"E; 25.07.–24.08.2017; TWT (on *Populustremula*); IS leg.; INS 33796. **Lääne-Virumaa** • ♂; near Lasila, forest patch No 79; 59°16'37"N, 26°13'22"E; 31.05.–28.06.2016; MT; OK leg.; INS 33782.

###### Comments.

Rather rare European species (Ježek 1984, 1994; Ježek and Omelková 2007; Kroča and Ježek 2022). Important species for nature conservation, assessed as endangered in the Czech Republic.

#### ﻿Paramormiini

**Paramormiina**

##### ﻿*6. Lepiseodina rothschildi

Taxon classificationAnimaliaDipteraPsychodidae

(Eaton, 1912)

BA383560-FC1C-51F4-8460-0EE5A0086DAC

###### Material examined.

**Saaremaa** • ♂; Ruhnu Island; 57°48'22"N, 23°15'08"E; 09.06.–10.07.2012; TWT (on dead *Piceaabies* (L.) H. Karst.); IS leg. **Pärnumaa** • ♂; Kihnu Island; 58°06'59"N, 23°59'38"E; 22.05.–11.06.2012; TWT (on old *Quercusrobur*); IS leg.; • ♂; Kihnu Island; 58°09'09"N, 24°00'32"E; 22.05.–11.06.2012; TWT (on *Quercusrobur*); IS leg.

###### Comments.

Rather rare European species (Oboňa et al. 2021; Jaume-Schinkel et al. 2022). Important species for nature conservation, assessed as nationally scarce in the Czech Republic.

##### ﻿7. Panimerus albifacies

Taxon classificationAnimaliaDipteraPsychodidae

(Tonnoir, 1919)

45ABD7DF-A182-5251-9CF2-4252D4A5A05A

###### Published record.

Salmela and Piirainen (2005): 304.

###### Material examined.

**Saaremaa** • ♂; Ruhnu Island; 57°48'20"N, 23°14'33"E; 07.06.–10.07.2012; TWT (on dead *Alnusglutinosa* (L.) Gaertn.); IS leg.

###### Comments.

European species (Ježek et al. 2021b; Jaume-Schinkel et al. 2023).

##### ﻿*8. Panimerus notabilis

Taxon classificationAnimaliaDipteraPsychodidae

(Eaton, 1893)

63840DCF-1D97-502F-8FD2-C12751F0A7B7

###### Material examined.

**Saaremaa** • ♂; Ruhnu Island; 57°48'20"N, 23°14'33"E; 07.08.–05.09.2012; TWT (on dead *Alnusglutinosa*); IS leg. **Pärnumaa** • ♂; Kihnu Island; 58°06'59"N, 23°59'38"E; 12.07.–09.08.2012; TWT (on old *Quercusrobur*); IS leg. **Tartumaa** • ♂; Vorbuse, Lokaatorite tee; 58°25'27"N, 26°35'47"E; 25.06.–14.07.2023; MT; OK leg.

###### Comments.

Common European species (Ježek et al. 2019; Jaume-Schinkel et al. 2023).

##### ﻿9. Parajungiella consors

Taxon classificationAnimaliaDipteraPsychodidae

(Eaton, 1893)

6CB1324E-A06B-58F6-B024-8E448CE4A232

###### Published record.

Salmela and Piirainen (2005): 304.

###### Comments.

Not common European species (Kroča and Ježek 2022).

##### ﻿*10. Parajungiella longicornis

Taxon classificationAnimaliaDipteraPsychodidae

(Tonnoir, 1919)

94324C87-E671-567E-9F7C-3454CB9BF656

###### Material examined.

**Pärnumaa** • ♂; Riimaru; 58°16'18"N, 25°11'02"E; 17.08.–31.08.2005; TWT (on *Populustremula*); IS leg. **Lääne-Virumaa** • ♂; Tudusoo NR; 59°04'47"N, 26°45'38"E; 28.05.–25.06.2017; TWT (on *Populustremula*); IS leg.; INS 33794.

###### Comments.

European and West-Siberian species (Ježek et al. 2020).

##### ﻿11. Parajungiella pseudolongicornis

Taxon classificationAnimaliaDipteraPsychodidae

(Wagner, 1975)

0B7D0EB0-929E-5C3F-8A05-FE4A80EAFCED

###### Published record.

Salmela and Piirainen (2005): 304.

###### Material examined.

**Lääne-Virumaa** • ♂; Suigu NR; 59°09'11"N, 26°49'10"E; 26.07.–29.08.2017; TWT (on dead *Populustremula*); IS leg.; INS 33779.

###### Comments.

Rare European species (Ježek and Omelková 2012; Kroča and Ježek 2022). Important species for nature conservation, assessed as critically endangered in the Czech Republic.

##### ﻿*12. Parajungiella serbica

Taxon classificationAnimaliaDipteraPsychodidae

(Krek, 1985)

64522BA5-E9A0-501E-825F-958B70238821

###### Material examined.

**Pärnumaa** • ♂; Riimaru; 58°16'19"N, 25°10'47"E; 02.06.–01.07.2006; TWT (on *Populustremula* with *Phellinustremulae* (Bondartsev) Bondartsev & N.P. Borisov); IS leg.

###### Comments.

Rather rare European and Transcaucasian species (Ježek et al. 2020). Important species for nature conservation, assessed as critically endangered in the Czech Republic.

##### ﻿13. Paramormia (Paramormia) polyascoidea

Taxon classificationAnimaliaDipteraPsychodidae

(Krek, 1971)

444A8250-EC96-58B7-8DE8-745809E078BE

###### Published record.

Salmela and Piirainen (2005): 304.

###### Comments.

European, West-Siberian and Transcaucasian species (Ježek et al. 2020, 2023b).

##### ﻿14. Peripsychoda auriculata

Taxon classificationAnimaliaDipteraPsychodidae

(Curtis, 1839)

FBC8DEE2-E7BE-59E7-8305-6995C4017C8D

###### Published record.

Salmela and Piirainen (2005): 304.

###### Material examined.

**Saaremaa** • ♂♂; Ruhnu Island; 57°48'15"N, 23°14'32"E; 07.06.–10.07.2012, 10.07.–07.08.2012; TWT (on *Acerplatanoides*); IS leg.

###### Comments.

European and Transcaucasian species (Ježek et al. 2020, 2023b; Morelli and Biscaccianti 2021).

##### ﻿15. Seoda carthusiana

Taxon classificationAnimaliaDipteraPsychodidae

(Vaillant, 1972)

043F0665-192D-5882-8F79-88D6BDFC4C72

###### Published record.

Salmela and Piirainen (2005): 304.

###### Comments.

European species (Kroča and Ježek 2022).

##### ﻿*16. Seoda gressica

Taxon classificationAnimaliaDipteraPsychodidae

(Vaillant, 1972)

0BFB3DB3-802A-5B6C-8249-DDE955B3ECF1

###### Material examined.

**Saaremaa** • ♂; Ruhnu Island; 57°47'59"N, 23°14'34"E; 26.05.–28.06.2011; TWT (on dead *Fraxinusexcelsior* L.); IS leg.; INS 33783. **Pärnumaa** • ♂♂; Kihnu Island; 58°07'49"N, 24°00'15"E; 22.05.–11.06.2012, 11.06.–12.07.2012; TWT (on old *Quercusrobur*); IS leg.

###### Comments.

Sporadically common European species (Ježek and Omelková 2012; Kroča and Ježek 2022).

##### ﻿*17. Seoda labeculosa

Taxon classificationAnimaliaDipteraPsychodidae

(Eaton, 1893)

4D2F57F0-716B-50AA-AFCB-76520850C3DA

###### Material examined.

**Lääne-Virumaa** • ♂♂; near Lasila, forest patch No 24; 59°16'27"N, 26°11'44"E; 30.05.–02.07.2015, 02.08.–24.08.2015; MT; OK leg.; • ♂; near Lasila, forest patch No 31; 59°15'48"N, 26°14'17"E; 30.05.–02.07.2015; MT; OK leg.; • ♂; near Lasila, forest patch No 50; 59°16'25"N, 26°14'02"E; 02.08.–24.08.2015; OK leg.; • ♂; near Lasila, forest patch No 79; 59°16'37"N, 26°13'22"E; 28.06.–28.07.2016; MT; OK leg.; INS 33831.

###### Comments.

Species known only from Europe (Ježek et al. 2019). Important for nature conservation, assessed as endangered in the Czech Republic.

#### ﻿Trichopsychodina

##### ﻿18. Feuerborniella obscura

Taxon classificationAnimaliaDipteraPsychodidae

(Tonnoir, 1919)

1A645F2D-1128-5AF6-80F2-C3B550FDFB26

###### Published record.

Salmela and Piirainen (2005): 304.

###### Material examined.

**Pärnumaa** • ♂♂; Riimaru; 58°16'19"N, 25°10'47"E; 11.06.–29.06.2005, 02.06.–01.07.2006; TWT (on *Populustremula* with *Phellinustremulae*); IS leg.; INS 33802, 33833.

###### Comments.

European and Transcaucasian species (Oboňa et al. 2019; Ježek et al. 2020, 2021a, b, 2023b).

##### ﻿*19. Philosepedon (Philosepedon) austriacum

Taxon classificationAnimaliaDipteraPsychodidae

Vaillant, 1974

6F85D669-FDE5-5D2B-9D49-D6D0172DAB81

###### Material examined.

**Lääne-Virumaa** • ♂; near Lasila, forest patch No 50; 59°16'25"N, 26°14'02"E; 28.07.–29.08.2016; MT; OK leg.; INS 33824.

###### Comments.

European species (Ježek et al. 2020; Kroča and Ježek 2022).

##### ﻿*20. Philosepedon (Philosepedon) dumosum

Taxon classificationAnimaliaDipteraPsychodidae

Omelková & Ježek, 2012

54C149A9-F7A0-5CBF-9669-97EA31FA23ED

###### Material examined.

**Pärnumaa** • ♂; Luitemaa NR near Võiste; 58°12'21"N, 24°29'36"E; 23.06.–25.07.2017; TWT (on burned *Pinussylvestris* L.); IS leg.; INS 33818.

###### Comments.

Species known only from the Czech Republic (Omelková and Ježek 2012a). Important for nature conservation, assessed as nationally scarce in the Czech Republic.

##### ﻿21. Philosepedon (Philosepedon) humerale

Taxon classificationAnimaliaDipteraPsychodidae

(Meigen, 1818)

F8F98D49-09D1-5400-82F7-3B2818E3987F

###### Published records.

Salmela and Piirainen (2005): 304.

###### Material examined.

**Hiiumaa** • ♂; Leigri NR; 58°53'51"N, 22°35'46"E; 19.07.–03.08.2013; TWT (on *Pinussylvestris*); IS leg. **Saaremaa** • ♂; Ruhnu Island, near church; 57°48'23"N, 23°14'41"E; 03.08.–11.09.2011; TWT (on old *Quercusrobur*); IS leg.; • ♂; Ruhnu Island; 57°47'55"N, 23°15'53"E; 24.05.–27.06.2011; TWT (on dead *Pinussylvestris*); IS leg.; • ♂♂; Ruhnu Island; 57°47'59"N, 23°14'34"E; 26.05.–28.06.2011, 28.06.–03.08.2011, 07.08.–05.09.2012; TWT (on dead *Fraxinusexcelsior*); IS leg.; • ♂♂; Ruhnu Island; 57°48'28"N, 23°14'32"E; 26.05.–28.06.2011, 28.06.–03.08.2011, 03.08.–11.09.2011; TWT (on dead *Fraxinusexcelsior*); IS leg.; • ♂; Ruhnu Island; 57°48'01"N, 23°14'38"E; 07.08.–05.09.2012; TWT (on *Acerplatanoides*); IS leg.; • ♂; Ruhnu Island; 57°48'08"N, 23°15'49"E; 06.08.–04.09.2012; TWT (on *Piceaabies* with *Fomitopsispinicola* (Sw.) P. Karst.); IS leg.; • ♂♂; Ruhnu Island; 57°48'09"N, 23°14'25"E; 07.06.–10.07.2012, 07.08.–05.09.2012; TWT (on *Fraxinusexcelsior*); IS leg.; • ♂; Ruhnu Island; 57°48'09"N, 23°14'29"E; 07.08.–05.09.2012; TWT (on *Corylusavellana* L.); IS leg.; • ♂♂; Ruhnu Island; 57°48'10"N, 23°14'24"E; 07.06.–10.07.2012, 10.07.–07.08.2012, 07.08.–05.09.2012; TWT (on *Fraxinusexcelsior*); IS leg.; • ♂♂; Ruhnu Island; 57°48'15"N, 23°14'32"E; 10.07.–07.08.2012, 07.08.–05.09.2012; TWT (on *Acerplatanoides*); IS leg.; • ♂; Ruhnu Island; 57°48'18"N, 23°14'27"E; 07.08.–05.09.2012; TWT (on *Quercusrobur*); IS leg.; • ♂; Ruhnu Island; 57°48'20"N, 23°14'33"E; 07.08.–05.09.2012; TWT (on *Alnusglutinosa*); IS leg.; • ♂; Ruhnu Island; 57°48'20"N, 23°15'30"E; 06.08.–04.09.2012; TWT (on dead *Piceaabies* with *Fomitopsispinicola*); IS leg.; • ♂; Ruhnu Island; 57°48'22"N, 23°15'08"E; 09.06.–10.07.2012; TWT (on dead *Piceaabies*); IS leg.; • ♂♂; Ruhnu Island; 57°48'02"N, 23°13'42"E; 07.06.–10.07.2012, 07.08.–05.09.2012; TWT (on old *Ulmuslaevis* Pallas); IS leg.; • ♂♂; Ruhnu Island; 57°48'27"N, 23°14'32"E; 09.06.–10.07.2012, 10.07.–07.08.2012, 07.08.–05.09.2012; TWT (on *Corylusavellana*); IS leg.; • ♂; Ruhnu Island; 57°47'54"N, 23°16'18"E; 06.08.–04.09.2012; TWT (on dead *Pinussylvestris*); IS leg. **Läänemaa** • ♂♂; Vormsi Island; 59°01'18"N, 23°08'00"E; 08.05.–04.06.2012, 03.08.–11.09.2012; TWT (on *Corylusavellana*); IS leg.; • ♂♂; Vormsi Island; 58°59'30"N, 23°11'34"E; 04.06.–06.07.2011, 06.07.–19.07.2011, 06.07.–03.08.2012; TWT (on *Quercusrobur*); IS leg.; • ♂♂; Vormsi Island; 58°59'26"N, 23°11'51"E; 08.05.–04.06.2012, 06.07.–03.08.2012; TWT (on *Salixfragilis* L.); IS leg.; • ♂; Vormsi Island; 58°58'11"N, 23°12'21"E; 08.05.–04.06.2012; TWT (on dead *Pinussylvestris*); IS leg.; • ♂; Vormsi Island; 59°01'20"N, 23°08'13"E; 09.05.–04.06.2012; TWT (on dead *Alnusglutinosa*); IS leg.; • ♂; Vormsi Island; 59°00'16"N, 23°13'49"E; 10.05.–04.06.2012; TWT (on *Pinussylvestris*); IS leg.; **Pärnumaa** • ♂; Kihnu Island; 58°06'59"N, 23°59'38"E; 02.07.–06.08.2011; TWT (on old *Quercusrobur*); IS leg.; • ♂; Kihnu Island; 58°08'28"N, 23°58'47"E; 01.07.–06.08.2011; TWT (on old *Pinussylvestris*); IS leg.; • ♂; Kihnu Island; 58°08'44"N, 23°58'14"E; 29.05.–01.07.2011; TWT (on old *Aesculushippocastanum*); IS leg.; • ♂; Kihnu Island; 58°08'44"N, 23°58'16"E; 06.08–13.09.2011; TWT (on old *Tiliacordata* Mill.); IS leg.; • ♂; Kihnu Island; 58°08'48"N, 23°58'17"E; 01.07.–06.08.2011; TWT (on dead *Fraxinusexcelsior*); IS leg.; • ♂; Kihnu Island, Lemsi; 58°07'49"N, 24°00'15"E; 06.08.–13.09.2011; TWT (on old *Quercusrobur*); IS leg.; • ♂♂; Kihnu Island; 58°06'59"N, 23°59'38"E; 22.05.–11.06.2012, 12.07.–09.08.2012; TWT (on old *Quercusrobur*); IS leg.; • ♂♂; Kihnu Island; 58°07'18"N, 23°58'25"E; 23.05.–11.06.2012, 11.06.–12.07.2012; TWT (on *Pinussylvestris*); IS leg.; • ♂♂; Kihnu Island; 58°07'49"N, 24°00'15"E; 22.05.–11.06.2012, 12.07.–09.08.2012; TWT (on old *Quercusrobur*); IS leg.; • ♂♂; Kihnu Island; 58°08'07"N, 23°58'20"E; 12.07.–09.08.2012, 09.08.–08.09.2012; TWT (on *Pinussylvestris*); IS leg.; • ♂♂; Kihnu Island; 58°08'28"N, 23°58'47"E; 23.05.–11.06.2012, 11.06.–12.07.2012, 12.07.–09.08.2012; TWT (on *Pinussylvestris*); IS leg.; • ♂♂; Kihnu Island; 58°08'44"N, 23°58'14"E; 23.05.–11.06.2012, 11.06.–12.07.2012; TWT (on old *Aesculushippocastanum*); IS leg.; • ♂; Kihnu Island; 58°09'09"N, 24°00'32"E; 11.06.–12.07.2012; TWT (on *Quercusrobur*); IS leg.; • ♂; Puhtu; 58°33'39.4"N, 23°33'07.9"E; 10.07.–13.08.2009; TWT (on dead *Piceaabies* with *Fomitopsispinicola*); IS leg.; • ♂; Puhtu; 58°33'25"N, 23°33'04"E; 27.05.–18.06.2009; TWT (on old *Pinussylvestris*); IS leg.; • ♂; Kalita NR; 58°04'16"N, 24°51'39"E; 25.07.–24.08.2017; TWT (on *Populustremula*); IS leg. **Lääne-Virumaa** • ♂; Lahemaa NP, Eru; 59°34'03"N, 25°51'53"E; 26.07.–26.08.2017; TWT (on burned *Piceaabies*); IS leg.; • ♂♂; Lahemaa NP, near Laukasoo; 59°28'49"N, 25°54'24"E; 23.05.–23.06.2017, 25.07.–26.08.2017; TWT (on dead *Populustremula*); IS leg.; • ♂♂; near Lasila, forest patch No 104; 59°16'43"N, 26°17'07"E; 12.–30.05.2015, 02.07.–02.08.2015, 20.09.–21.10.2015, 31.05.–28.06.2016; MT; OK leg.; • ♂♂; near Lasila, forest patch No 24; 59°16'27"N, 26°11'44"E; 12.–30.05.2015, 30.05.–02.07.2015, 02.07.–02.08.2015, 02.–24.08.2015, 24.08.–20.09.2015, 20.09.–21.10.2015, 31.05.–28.06.2016, 05.–31.05.2016; MT; OK leg.; • ♂♂; near Lasila, forest patch No 31; 59°15'48"N, 26°14'17"E; 12.–30.05.2015, 30.05.–02.07.2015, 02.07.–02.08.2015, 02.–24.08.2015, 24.08.–20.09.2015, 20.09.–21.10.2015, 28.06.–28.07.2016; MT; OK leg.; • ♂♂; near Lasila, forest patch No 50; 59°16'25"N, 26°14'02"E; 12.–30.05.2015, 02.–24.08.2015, 31.05.–28.06.2016, 28.06.–28.07.2016, 29.08.–26.09.2016; MT; OK leg.; • ♂♂; near Lasila, forest patch No 52; 59°16'20"N, 26°13'52"E; 31.05.–28.06.2016, 28.06.–28.07.2016, 28.07.–29.08.2016, 29.08.–26.09.2016; MT; OK leg.; • ♂♂; near Lasila, forest patch No 6; 59°15'41"N, 26°12'32"E; 12.–30.05.2015, 30.05.–02.07.2015, 02.07.–02.08.2015, 02.–24.08.2015, 24.08.–20.09.2015, 20.09.–21.10.2015, 31.05.–28.06.2016, 28.07.–29.08.2016, 29.08.–26.09.2016, 26.09.–27.10.2016; MT; OK leg.; • ♂♂; near Lasila, forest patch No 7; 59°15'33"N, 26°12'41"E; 12.–30.05.2015, 02.07.–02.08.2015, 02.–24.08.2015; MT; OK leg.; • ♂♂; near Lasila, forest patch No 75; 59°16'49"N, 26°13'05"E; 30.05.–02.07.2015, 02.07.–02.08.2015, 20.09.–21.10.2015, 05.–31.05.2016, 28.07.–29.08.2016; MT; OK leg.; • ♂♂; near Lasila, forest patch No 79; 59°16'37"N, 26°13'22"E; 12.–30.05.2015, 30.05.–02.07.2015, 02.07.–02.08.2015, 02.–24.08.2015, 20.09.–21.10.2015, 05.–31.05.2016, 31.05.–28.06.2016, 28.06.–28.07.2016, 28.07.–29.08.2016, 29.08.–26.09.2016; MT; OK leg. **Ida-Virumaa** • ♂; Muraka NR; 59°05'22"N, 27°09'18"E; 15.06.–30.06.2015; TWT (on dead *Piceaabies*); IS leg. **Tartumaa** • ♂; Vorbuse, Lokaatorite tee; 58°25'27"N, 26°35'47"E; 10.–25.06.2023; MT; OK leg.; • ♂; Elva-Vitipalu PL; 58°10'49"N, 26°25'14"E; 13.06.–29.06.2015; TWT (on dead *Piceaabies*); IS leg. **Valgamaa** • ♂♂; Soontaga; 58°00'04"N, 26°05'11"E; 07.05.–05.06.2015, 05.06.–06.07.2015, 03.–31.08.2015; TWT (on dead *Betulapendula* with *Fomesfomentarius* (L.) Fr.); IS leg.; • ♂♂; Soontaga; 58°00'04"N, 26°05'15"E; 07.05.–05.06.2015, 05.06.–06.07.2015, 06.07.–03.08.2015; TWT (on dead *Betulapendula*); IS leg.; • ♂♂; Soontaga; 58°00'01"N, 26°05'10"E; 19.05.–05.06.2015, 05.06.–06.07.2015, 06.07.–03.08.2015, 03.–31.08.2015; TWT (on dead *Populustremula*); IS leg.; • ♂♂; Soontaga; 58°00'03"N, 26°05'31"E; 11.05.–05.06.2015, 05.06–06.07.2015, 06.07.–03.08.2015; TWT (on *Acerplatanoides*); IS leg.; • ♂; Koiva wooded meadow; 57°41'21"N, 26°11'08"E; 14.05.–03.06.2013; TWT (on dead *Quercusrobur*); IS leg.; • ♂; Koiva wooded meadow; 57°41'19"N, 26°10'59"E; 01.08.–17.08.2013; TWT (on dead *Quercusrobur*); IS leg.; • ♂; Koivakonnu; 57°35'28"N, 26°19'21"E; 01.08.–17.08.2013; TWT (on dead *Quercusrobur*); IS leg. **Võrumaa** • ♂; Haanja NP; 57°44'07"N, 27°03'50"E; 18.05.–20.06.2017; TWT (on dead *Populustremula*); IS leg.; • ♂; Haanja NP; 57°44'08"N, 27°03'46"E; 20.06.–23.07.2017; TWT (on dead *Betulapendula* with *Fomesfomentarius*); IS leg.; • ♂; Piusa PL; 57°47'28"N, 27°21'57"E; 18.05.–19.06.2017, 19.06.–23.07.2017; TWT (on burned *Pinussylvestris*); IS leg.

###### Comments.

Very common European species (Ježek et al. 2020).

##### ﻿*22. Philosepedon (Philothreticus) soljani

Taxon classificationAnimaliaDipteraPsychodidae

Krek, 1971

A7153CE5-15BC-5684-B600-58175BBF7C7C

###### Material examined.

**Lääne-Virumaa** • ♂; near Lasila, forest patch No 50; 59°16'25"N, 26°14'02"E; 28.07.–29.08.2016; MT; OK leg.

###### Comments.

European species (Ježek et al. 2017; Kroča and Ježek 2022). Important for nature conservation, assessed as nationally scarce in the Czech Republic.

##### ﻿*23. Philosepedon (Trichosepedon) balkanicum

Taxon classificationAnimaliaDipteraPsychodidae

Krek, 1970

DB7E81F1-32E1-51EE-BB66-ED085143CA9D

###### Material examined.

**Hiiumaa** • ♂; Leigri NR; 58°53'00"N, 22°35'25"E; 05.06.–18.06.2013; TWT (on dead *Piceaabies*); IS leg. **Saaremaa** • ♂; Ruhnu Island; 57°48'23"N, 23°14'41"E; 03.08.–11.09.2011; TWT (on *Quercusrobur*); IS leg.; • ♂; Ruhnu Island; 57°47'54"N, 23°15'26"E; 06.08.–04.09.2012; TWT (on *Pinussylvestris*); IS leg. **Läänemaa** • ♂; Vormsi Island; 59°01'01"N, 23°12'18"E; 03.08.–01.09.2012; TWT (on *Pinussylvestris*); IS leg.; • ♂♂; Vormsi Island; 59°01'22"N, 23°12'12"E; 19.08.–04.09.2011, 03.08.–01.09.2012; TWT (dead *Piceaabies* with *Fomitopsispinicola*); IS leg. **Pärnumaa** • ♂; Riimaru; 58°16'18"N, 25°11'02"E; 02.06.–01.07.2006; TWT (on *Populustremula*); IS leg.; • ♂♂; Riimaru; 58°16'18"N, 25°11'02"E; 11.–29.06.2005, 17.–31.08.2005; TWT (on *Populustremula*); IS leg.; INS 33806; • ♂; Kalita NR; 58°04'15"N, 24°51'37"E; 25.07.–24.08.2017; TWT (on *Populustremula*); IS leg.; • ♂; Luitemaa NR near Võiste; 58°12'21"N, 24°29'34"E; 20.05.–23.06.2017; TWT (on burned *Pinussylvestris*); IS leg.; • ♂; Kihnu Island; 58°07'49"N, 24°00'15"E; 12.07.–09.08.2012; TWT (on *Quercusrobur*); IS leg. **Viljandimaa** • ♂; Muti PL; 58°08'25"N, 25°40'50"E; 21.06.–24.07.2017; TWT (on dead *Betulapendula*); IS leg. **Ida-Virumaa** • ♂; Muraka NR S of Virunurme; 59°09'52"N, 27°00'48"E; 26.05.–25.06.2017; TWT (on dead *Populustremula*); IS leg.; • ♂; Muraka NR; 59°05'22"N, 27°09'18"E; 30.05.–15.06.2015; TWT (on dead *Piceaabies*); IS leg.; • ♂; Agusalu NR; 59°02'16"N, 27°39'35"E; 30.05.–15.06.2015; TWT (on dead *Pinussylvestris*); IS leg. **Tartumaa** • ♂; Padakõrve NR; 58°35'07"N, 27°01'09"E; 30.05.–15.06.2015; TWT (on dead *Pinussylvestris*); IS leg.; • ♂; Järvselja NR; 58°16'52"N, 27°19'27"E; 17.05.–19.06.2017; TWT (on dead *Populustremula*); IS leg. **Põlvamaa** • ♂; Ihamaru NR; 58°05'52"N, 26°55'33"E; 29.05.–13.06.2015; TWT (on dead *Piceaabies*); IS leg.

###### Comments.

European and Transcaucasian species (Kvifte 2019; Ježek et al. 2023b).

##### ﻿*24. Trichopsychoda hirtella

Taxon classificationAnimaliaDipteraPsychodidae

(Tonnoir, 1919)

CCF44A17-EF0D-583B-91BE-9ABD4C267C8F

###### Material examined.

**Saaremaa** • ♂♂; Ruhnu Island, near Korsi farm; 57°48'28"N, 23°14'32"E; 26.05.–28.06.2011, 28.06.–03.08.2011; TWT (on dead *Fraxinusexcelsior*); IS leg.; INS 33832; • ♂; Ruhnu Island; 57°48'08"N, 23°15'49"E; 06.08.–04.09.2012; TWT (on dead *Piceaabies* with *Fomitopsispinicola*); IS leg.; • ♂; Ruhnu Island; 57°48'09"N, 23°14'25"E; 07.08.–05.09.2012; TWT (on *Fraxinusexcelsior*); IS leg.; • ♂; Ruhnu Island; 57°48'10"N, 23°14'24"E; 07.08.–05.09.2012; TWT (on *Fraxinusexcelsior*); IS leg.; • ♂; Ruhnu Island; 57°48'15"N, 23°14'32"E;10.07.–07.08.2012; TWT (on *Acerplatanoides*); IS leg.; • ♂; Ruhnu Island; 57°48'18"N, 23°14'27"E; 07.08.–05.09.2012; TWT (on *Quercusrobur*); IS leg.; • ♂; Ruhnu Island, near Korsi farm; 57°48'27"N, 23°14'32"E; 09.06.–10.07.2012; TWT (on *Corylusavellana*); IS leg.; • ♂; Pihtla; 21.07.2016; hand picked; M. Oras leg. **Läänemaa** • ♂; Kullamaa; 23.07.2016; sweep net; K. Sammet leg. **Pärnumaa** • ♂♂; Riimaru; 58°16'19"N, 25°10'47"E; 29.06.–13.07.2005, 02.06.–01.07.2006; TWT (on *Populustremula* with *Phellinustremulae*); IS leg.; INS 33788. **Lääne-Virumaa** • ♂; near Lasila, forest patch No 31; 59°15'48"N, 26°14'17"E; 02.07.–02.08.2015; MT; OK leg.; • ♂; near Lasila, forest patch No 6; 59°15'41"N, 26°12'32"E; 28.07.–29.08.2016; MT; OK leg. **Tartumaa** • ♂; Vapramäe; 15.07.2016; sweep net; OK leg. **Valgamaa** • ♂; Soontaga; 58°00'03"N, 26°05'31"E; 05.06.–06.07.2015; TWT (on *Acerplatanoides*); IS leg.; • ♂; Soontaga; 58°00'04"N, 26°05'11"E; 05.06.–06.07.2015; TWT (on dead *Betulapendula* with *Fomesfomentarius*); IS leg.; • ♂; Koivakonnu; 57°35'28"N, 26°19'21"E; 16.06.–02.07.2013; TWT (on dead *Quercusrobur*); IS leg.

###### Comments.

European and Transcaucasian species (Ježek et al. 2020, 2023b; Morelli and Biscaccianti 2021).

#### ﻿Psychodini

##### ﻿*25. Chodopsycha buxtoni

Taxon classificationAnimaliaDipteraPsychodidae

(Withers, 1988)

9BB0E290-AD51-5944-814A-6AABAB22D460

###### Material examined.

**Lääne-Virumaa** • ♂; near Lasila, forest patch No 50; 59°16'25"N, 26°14'02"E; 28.07.–29.08.2016; MT; OK leg.; INS 33825. **Valgamaa** • ♂; Koivakonnu; 57°35'28"N, 26°19'21"E; 17.08.–01.09.2013; TWT (on dead *Quercusrobur*); IS leg.; INS 33834.

###### Comments.

Not common European and Transcaucasian species (Ježek et al. 2023b). Important for nature conservation, assessed as nationally scarce in the Czech Republic.

##### ﻿*26. Chodopsycha lobata

Taxon classificationAnimaliaDipteraPsychodidae

(Tonnoir, 1940)

E615DAEA-A17C-5AEF-85B1-F5CEA4B6E3F0

###### Material examined.

**Lääne-Virumaa** • ♀; near Lasila, forest patch No 50; 59°16'25"N, 26°14'02"E; 28.07.–29.08.2016; MT; OK leg.; INS 33823; • ♂; Tudusoo NR; 59°05'01"N, 26°45'54"E; 27.07.–29.08.2017; TWT (on dead *Populustremula*); IS leg.; INS 33828.

###### Comments.

Common European and Transcaucasian species (Ježek et al. 2020, 2021a, b).

##### ﻿27. Logima albipennis

Taxon classificationAnimaliaDipteraPsychodidae

(Zetterstedt, 1850)

1DB67DB6-5B3D-5F5B-9A19-58EE0CE54748

###### Published record.

Salmela and Piirainen (2005): 304.

###### Material examined.

**Pärnumaa** • ♂♂; Riimaru; 58°16'18"N, 25°11'02"E; 03.08.–17.08.2005, 31.07.–02.09.2006; TWT (on dead *Betulapendula* with *Fomesfomentarius*); IS leg.; • ♂; Kalita NR; 58°04'15"N, 24°51'37"E; 25.07.–24.08.2017; TWT (on *Populustremula*); IS leg.; INS 33795.

###### Comments.

Cosmopolitan species (Ježek et al. 2021a, 2023a).

##### ﻿*28. Logima satchelli

Taxon classificationAnimaliaDipteraPsychodidae

(Quate, 1955)

399800C7-2E1E-5888-B1A6-D64607B69961

###### Material examined.

**Saaremaa** • ♂; Ruhnu Island; 57°47'55"N, 23°15'53"E; 24.05.–27.06.2011; TWT (on dead *Pinussylvestris*); IS leg.; • ♂; Ruhnu Island, near Korsi farm; 57°48'28"N, 23°14'32"E; 26.05.–28.06.2011; TWT (on dead *Fraxinusexcelsior*); IS leg.; • ♂; Ruhnu Island; 57°48'09"N, 23°14'25"E; 10.07.–07.08.2012; TWT (on *Fraxinusexcelsior*); IS leg.; • ♂; Ruhnu Island; 57°48'09"N, 23°14'29"E; 07.06.–10.07.2012; TWT (on *Corylusavellana*); IS leg.; • ♂; Ruhnu Island; 57°48'18"N, 23°14'27"E; 07.08.–05.09.2012; TWT (on *Quercusrobur*); IS leg.; • ♂♂; Ruhnu Island, Holma; 57°48'02"N, 23°13'42"E; 29.06.–02.08.2011, 07.06.–10.07.2012; TWT (on old *Ulmuslaevis*); IS leg.; • ♂♂; Ruhnu Island, Korsi farm; 57°48'27"N, 23°14'32"E; 09.06.–10.07.2012, 10.07.–07.08.2012; TWT (on *Corylusavellana*); IS leg. **Läänemaa** • ♂; Puise; 58°47'45"N, 23°31'22"E; 17.06.–11.07.2009; TWT (on old *Betulapendula*); IS leg.; • ♂; Vormsi Island; 58°58'33"N, 23°12'10"E; 03.08.–01.09.2012; TWT (on dead *Betulapendula* with *Fomesfomentarius*); IS leg.; • ♂; Vormsi Island; 59°01'22"N, 23°12'12"E; 03.08.–01.09.2012; TWT (on dead *Piceaabies* with *Fomitopsispinicola*); IS leg.; • ♂; Vormsi Island; 58°59'26"N, 23°11'51"E; 08.05.–04.06.2012; TWT (on *Salixfragilis*); IS leg. **Pärnumaa** • ♂; Kihnu Island; 58°08'07"N, 23°58'20"E; 24.05.–11.06.2012; TWT (on *Pinussylvestris* with *Phellinuspini*); IS leg.; • ♂; Matsalu NP; 58°43'52"N, 23°42'54"E; 17.06.–11.07.2009; TWT (on dead *Betulapendula* with *Fomesfomentarius*); IS leg.; • ♂; Riimaru; 58°16'18"N, 25°11'02"E; 19.06.–03.08.2007; TWT (on *Populustremula*); IS leg.; • ♂; Riimaru; 58°16'19"N, 25°10'47"E; 17.–31.08.2005; TWT (on *Populustremula* with *Phellinustremulae*); IS leg.; • ♂; Riimaru; 58°16'18"N, 25°11'02"E; 31.07.–02.09.2006; TWT (on dead *Betulapendula* with *Fomesfomentarius*); IS leg.; • ♂; Luitemaa NR near Võiste; 58°12'21"N, 24°29'36"E; 23.06.–25.07.2017; TWT (on burned *Pinussylvestris*); IS leg. **Viljandimaa** • ♂; Muti PL; 58°08'25"N, 25°40'50"E; 19.05.–21.06.2017; TWT (on dead *Betulapendula* with *Fomesfomentarius*); IS leg. **Harjumaa** • ♂♂; Mähuste near Koitjärve; 59°24'55"N, 25°36'45"E; 01.07.–16.07.2015, 16.–30.07.2015, 15.08.–03.09.2015; TWT (on dead *Betulapendula*); IS leg.; • ♂♂; Põhja-Kõrvemaa NR; 59°25'30"N, 25°39'11"E; 15.05.–01.06.2015, 01.–16.06.2015, 16.06.–01.07.2015; TWT (on dead *Pinussylvestris*); IS leg. **Lääne-Virumaa** • ♂; near Lasila, forest patch No 6; 59°15'41"N, 26°12'32"E; 31.05.–28.06.2016; MT; OK leg.; • ♂; near Lasila, forest patch No 7; 59°15'33"N, 26°12'41"E; 26.09.–27.10.2016; MT; OK leg.; • ♂; Suigu NR; 59°08'59"N, 26°49'11"E; 25.06.–26.07.2017; TWT (on *Populustremula*); IS leg.; • ♀; Suigu NR; 59°09'11"N, 26°49'10"E; 25.06.–26.07.2017; TWT (on dead *Populustremula*); IS leg. **Ida-Virumaa** • ♂; Kautvere near Oonurme; 59°08'23"N, 26°57'10"E; 26.07.–28.08.2017; TWT (on dead *Populustremula*); IS leg.; • ♂♂; Kurtna PL; 59°18'09"N, 27°34'08"E; 30.05.–15.06.2015, 15.–29.07.2015; TWT (on dead *Pinussylvestris*); IS leg.; • ♂; Kurtna PL; 59°18'22"N, 27°33'53"E; 29.07.–14.08.2015; TWT (on dead *Pinussylvestris*); IS leg.; • ♂; Agusalu NR; 59°04'16"N, 27°37'40"E; 14.–30.05.2015; TWT (on dead *Betulapendula* with *Fomesfomentarius*); IS leg.; • ♂; Agusalu NR; 59°02'17"N, 27°39'40"E; 29.07.–14.08.2015; TWT (on dead *Pinussylvestris*); IS leg.; • ♂♂; Agusalu NR; 59°02'16"N, 27°39'35"E; 01.–14.05.2015, 30.05.–15.06.2015, 14.08.–01.09.2015; TWT (on dead *Pinussylvestris*); IS leg.; • ♂; Agusalu NR; 59°03'56"N, 27°37'37"E; 30.04.–14.05.2015; TWT (on dead *Betulapendula*); IS leg.; • ♂; Agusalu NR; 59°04'16"N, 27°37'40"E; 30.04.–14.05.2015; TWT (on dead *Betulapendula* with *Fomesfomentarius*); IS leg.; • ♂♂; Agusalu NR; 59°07'10"N, 27°34'39"E; 30.04.–14.05.2015, 14.08.–01.09.2015; TWT (on dead *Betulapendula* with *Fomesfomentarius*); IS leg.; • ♂♂; Agusalu NR; 59°02'15"N, 27°39'42"E; 15.–30.06.2015, 14.08.–01.09.2015; TWT (on dead *Betulapendula*); IS leg. **Tartumaa** • ♂♂; Padakõrve NR; 58°35'07"N, 27°01'09"E; 30.04.–14.05.2015, 14.05.–30.05.2015, 30.05.–15.06.2015, 15.–30.06.2015, 14.08.–01.09.2015; TWT (on dead *Pinussylvestris*); IS leg.; • ♂♂; Rootsiküla; 58°37'14"N, 27°10'47"E; 10.05.–06.06.2009, 02.–18.08.2009; TWT (on *Salixcaprea* L.); IS leg.; • ♂; Vorbuse, Lokaatorite tee; 58°25'27"N, 26°35'47"E; 01.–15.10.2023; MT; OK leg.; • ♂; Välgi; 58°33'48"N, 26°52'38"E; 15.–29.07.2015; TWT (on *Populustremula*); IS leg.; • ♂; Peipsiveere NR; 58°17'22"N, 27°08'59"E; 19.06.–22.07.2017; TWT (on dead *Populustremula*); IS leg.; • ♂; Elva-Vitipalu PL; 58°10'49"N, 26°25'14"E; 27.04.–13.05.2015; TWT (on dead *Piceaabies*); IS leg. **Põlvamaa** • ♂; Ihamaru NR; 58°06'09"N, 26°55'59"E; 27.04.–13.05.2015; TWT (on dead *Betulapendula* with *Fomesfomentarius*); IS leg. **Valgamaa** • ♂♂; Soontaga; 58°00'03"N, 26°05'31"E; 05.06.–06.07.2015, 06.07.–03.08.2015; TWT (on *Acerplatanoides*); IS leg.; • ♂; Soontaga; 58°00'04"N, 26°05'11"E; 05.06.–06.07.2015; TWT (on dead *Betulapendula* with *Fomesfomentarius*); IS leg.; • ♂; Soontaga; 58°00'01"N, 26°05'10"E; 05.06.–06.07.2015; TWT (on *Populustremula*); IS leg.; • ♂♂; Soontaga; 58°00'04"N, 26°05'15"E; 05.06.–06.07.2015, 06.07.–03.08.2015; TWT (on dead *Betulapendula*); IS leg.; • ♂; Tündre NR; 57°56'59"N, 25°37'51"E; 21.06.–24.07.2017; TWT (on burned *Piceaabies*); IS leg.; • ♂; Koiva wooded meadow; 57°41'21"N, 26°10'54"E; 17.08.–01.09.2013; TWT (on dead *Salixcaprea*); IS leg.; • ♂; Koiva wooded meadow; 57°41'19"N, 26°10'59"E; 17.07.–01.08.2013; TWT (on dead *Quercusrobur*); IS leg.; • ♂; Koivakonnu; 57°35'27"N, 26°19'40"E; 16.06.–02.07.2013; TWT (on dead *Betulapendula* with *Fomesfomentarius*); IS leg.; • ♂♂; Koivakonnu; 57°35'28"N, 26°19'21"E; 16.06.–02.07.2013, 01.–17.08.2013; TWT (on dead *Quercusrobur*); IS leg.

###### Comments.

Holarctic species (Ježek et al. 2020, 2023a, b).

##### ﻿*29. Logima sigma

Taxon classificationAnimaliaDipteraPsychodidae

(Kincaid, 1899)

B1B53BB6-C548-52B3-80FC-9D5D38330F36

###### Material examined.

**Tartumaa** • ♀; Rootsiküla; 58°37'14"N, 27°10'47"E; 24.06.–15.07.2009; TWT (on dead *Salixcaprea*); IS leg.; INS 33804.

###### Comments.

Probably cosmopolitan species (Oboňa and Kozánek 2018; Ježek et al. 2021a).

##### ﻿30. Psychoda phalaenoides

Taxon classificationAnimaliaDipteraPsychodidae

(Linnaeus, 1758)

0D4FBF6E-AF95-541C-BE1F-248B65DB3B87

###### Published records.

Remm (1956): 234; Salmela and Piirainen (2005): 304.

###### Material examined.

**Saaremaa** • ♂; Ruhnu Island, Holma; 57°48'02"N, 23°13'42"E; 07.08.–05.09.2012; TWT (on old *Ulmuslaevis*); IS leg.; • ♂; Ruhnu Island, Korsi farm; 57°48'27"N, 23°14'32"E; 07.08.–05.09.2012; TWT (on *Corylusavellana*); IS leg.; • ♂; Ruhnu Island, Limo seashore; 57°47'37"N, 23°16'17"E; 06.08.–04.09.2012; TWT (on *Pinussylvestris*); IS leg. **Läänemaa** • ♂; Vormsi Island; 59°01'18"N, 23°08'00"E; 08.05.–04.06.2012; TWT (on *Corylusavellana*); IS leg. **Pärnumaa** • ♂; Matsalu NP; 58°42'41"N, 23°41'19"E;11.07.–14.08.2009; TWT (on *Populustremula*); IS leg.; • ♂; Matsalu NP; 58°42'52"N, 23°41'23"E; 17.06.–11.07.2009; TWT (on dead *Populustremula*); IS leg.; • ♂♂; Riimaru; 58°16'18"N, 25°11'02"E; 02.06.–01.07.2006, 19.06.–03.08.2007; TWT (on dead *Populustremula*); IS leg.; • ♂; Riimaru; 58°16'19"N, 25°10'47"E; 02.06.–01.07.2006; TWT (on *Populustremula* with *Phellinustremulae*); IS leg.; • ♂; Kalita NR; 58°04'16"N, 24°51'39"E; 22.06.–25.07.2017; TWT (on *Populustremula*); IS leg. **Viljandimaa** • ♂♂; Muti PL; 58°08'25"N, 25°40'50"E; 21.06.–24.07.2017, 24.07.–24.08.2017; TWT (on dead *Betulapendula* with *Fomesfomentarius*); IS leg. **Harjumaa** • ♂; Mähuste near Koitjärve; 59°24'55"N, 25°36'45"E; 16.07.–30.07.2015; TWT (on dead *Betulapendula*); IS leg. **Lääne-Virumaa** • ♂; Sakusaare; 59°28'58"N, 26°01'54"E; 25.07.–26.08.2017; TWT (dead *Pinussylvestris*); IS leg.; • ♂; near Lasila, forest patch No 104; 59°16'43"N, 26°17'07"E; 02.07.–02.08.2015; MT; OK leg.; • ♂; near Lasila, forest patch No 31; 59°15'48"N, 26°14'17"E; 28.07.–29.08.2016; MT; OK leg.; • ♂♂; near Lasila, forest patch No 50; 59°16'25"N, 26°14'02"E; 28.06.–28.07.2016, 29.08.–26.09.2016, 26.09.–27.10.2016; MT; OK leg.; • ♂♂; near Lasila, forest patch No 6; 59°15'41"N, 26°12'32"E; 28.07.–29.08.2016, 26.09.–27.10.2016; MT; OK leg.; • ♂; near Lasila, forest patch No 7; 59°15'33"N, 26°12'41"E; 02.–24.08.2015; MT; OK leg.; • ♂♂; near Lasila, forest patch No 75; 59°16'49"N, 26°13'05"E; 02.07.–02.08.2015, 02.–24.08.2015, 20.09.–21.10.2015, 28.07.–29.08.2016; MT; OK leg.; • ♂♂; near Lasila, forest patch No 79; 59°16'37"N, 26°13'22"E; 02.–24.08.2015, 24.08.–20.09.2015, 05.–31.05.2016; MT; OK leg.; • ♀; Tudusoo NR; 59°04'52"N, 26°46'16"E; 26.07.–29.08.2017; TWT (on dead *Populustremula*); IS leg. **Ida-Virumaa** • ♂; Kautvere near Oonurme; 59°08'23"N, 26°57'10"E; 26.05.–25.06.2017; TWT (on dead *Populustremula*); IS leg.; • ♂; Muraka NR, S of Virunurm; 59°09'52"N, 27°00'48"E; 26.07.–28.08.2017; TWT (on dead *Populustremula*); IS leg.; • ♂; Muraka NR; 59°05'22"N, 27°09'18"E; 30.04.–14.05.2015; TWT (on dead *Piceaabies*); IS leg.; • ♂; Kurtna PL; 59°18'09"N, 27°34'08"E; 15.–29.07.2015; TWT (on dead *Pinussylvestris*); IS leg.; • ♂; Kurtna PL; 59°18'22"N, 27°33'53"E; 15.–30.06.2015, 29.07.–14.08.2015; TWT (on dead *Pinussylvestris*); IS leg.; • ♂♂; Agusalu NR; 59°02'16"N, 27°39'35"E; 01.–14.05.2015, 14.08.–01.09.2015; TWT (on dead *Pinussylvestris*); IS leg.; • ♂; Agusalu NR; 59°02'17"N, 27°39'40"E; 15.–29.07.2015; TWT (on dead *Pinussylvestris*); IS leg.; • ♂; Agusalu NR; 59°04'16"N, 27°37'40"E; 15.–30.06.2015; TWT (on dead *Betulapendula* with *Fomesfomentarius*); IS leg.; • ♂; Agusalu NR; 59°07'10"N, 27°34'39"E; 29.07.–14.08.2015; TWT (on dead *Betulapendula* with *Fomesfomentarius*); IS leg. **Tartumaa** • ♂; Välgi NR; 58°33'48"N, 26°52'38"E; 30.04.–14.05.2015; TWT (on *Populustremula*); IS leg.; • ♂; Padakõrve NR; 58°35'07"N, 27°01'09"E; 30.04.–14.05.2015; TWT (on dead *Pinussylvestris*); IS leg.; • ♂; Rootsiküla; 58°37'14"N, 27°10'47"E; 06.–24.06.2009; TWT (on dead *Salixcaprea*); IS leg.; • ♂♂; Vorbuse, Lokaatorite tee; 58°25'27"N, 26°35'47"E; 30.04.–14.05.2023, 14.07.–02.08.2023, 02.08.–16.08.2023, 16.08.–30.08.2023, 30.08.–16.09.2023, 18.09.–01.10.2023, 01.10.–15.10.2023; MT; OK leg. **Valgamaa** • ♂; Soontaga; 58°00'03"N, 26°05'31"E; 05.06.–06.07.2015; TWT (on *Acerplatanoides*); IS leg.; • ♂♂; Soontaga NR; 58°00'23"N, 26°03'19"E; 27.04.–13.05.2015, 29.05.–13.06.2015, 14.–28.07.2015; TWT (on dead *Piceaabies*); IS leg.; • ♂; Koiva wooded meadow; 57°41'21"N, 26°11'08"E; 01.–17.08.2013; TWT (on dead *Quercusrobur*); IS leg.; • ♂♂; Koivakonnu; 57°35'27"N, 26°19'40"E; 14.05.–03.06.2013, 03.–16.06.2013, 16.06.–02.07.2013, 02.–17.07.2013; TWT (on dead *Betulapendula* with *Fomesfomentarius*); IS leg.; • ♂♂; Koivakonnu; 57°35'28"N, 26°19'21"E; 14.05.–03.06.2013, 16.06.–02.07.2013, 17.08.–01.09.2013; TWT (on dead *Quercusrobur*); IS leg. **Võrumaa** • ♂; Piusa PL; 57°47'28"N, 27°21'57"E; 18.05.–19.06.2017; TWT (on burned *Pinussylvestris*); IS leg.; • ♂; Piusa PL; 57°47'33"N, 27°21'57"E; 19.06.–23.07.2017; TWT (on burned *Pinussylvestris*); IS leg.

###### Comments.

Holarctic species (Wagner 2018; Ježek et al. 2020, 2021a, b).

##### ﻿*31. Psychodocha cinerea

Taxon classificationAnimaliaDipteraPsychodidae

(Banks, 1894)

78368E1D-037F-5BE2-B54D-80DBA1243B95

Fig. 3A

###### Material examined.

**Saaremaa** • ♂; Ruhnu Island; 57°47'46"N, 23°16'17"E; 06.08.–04.09.2012; TWT (on *Pinussylvestris*); IS leg.; • ♂; Ruhnu Island; 57°47'54"N, 23°15'26"E; 06.08.–04.09.2012; TWT (on *Pinussylvestris*); IS leg.; • ♂; Ruhnu Island; 57°48'01"N, 23°14'38"E; 07.08.–05.09.2012; TWT (on *Acerplatanoides*); IS leg.; • ♂; Ruhnu Island; 57°48'06"N, 23°13'58"E; 07.08.–05.09.2012; TWT (on *Sorbusintermedia* with *Laetiporussulphureus* (Bull.) Murrill); IS leg.; • ♂; Ruhnu Island; 57°48'09"N, 23°14'29"E; 07.08.–05.09.2012; TWT (on *Corylusavellana*); IS leg.; • ♂; Ruhnu Island; 57°48'10"N, 23°14'24"E; 10.07.–07.08.2012; TWT (on *Fraxinusexcelsior*); IS leg.; • ♂; Ruhnu Island; 57°48'15"N, 23°14'32"E; 07.08.–05.09.2012; TWT (on *Acerplatanoides*); IS leg.; • ♂; Ruhnu Island; 57°48'16"N, 23°16'06"E; 06.08.–04.09.2012; TWT (on dead *Pinussylvestris*); IS leg.; • ♂; Ruhnu Island; 57°48'20"N, 23°14'33"E; 07.08.–05.09.2012; TWT (on dead *Alnusglutinosa*); IS leg.; • ♂; Ruhnu Island; 57°48'24"N, 23°15'16"E; 07.08.–05.09.2012; TWT (on dead *Salixcaprea*); IS leg.; • ♂♂; Ruhnu Island, Korsi farm; 57°48'27"N, 23°14'32"E; 10.07.–07.08.2012; 07.08.–05.09.2012; TWT (on *Corylusavellana*); IS leg.; • ♂; Ruhnu Island, Limo seashore; 57°47'54"N, 23°16'18"E; 06.08.–04.09.2012; TWT (on dead *Pinussylvestris*); IS leg.; • ♂; Ruhnu Island; 57°48'08"N, 23°15'49"E; 06.08.–04.09.2012; TWT (on dead *Piceaabies* with *Fomitopsispinicola*); IS leg. **Läänemaa** • ♂; Vormsi Island; 59°00'17"N, 23°10'58"E; 03.08.–01.09.2012; TWT (on dead *Piceaabies*); IS leg.; • ♂♂; Vormsi Island; 59°01'20"N, 23°08'13"E; 09.05.–04.06.2012, 03.08.–01.09.2012; TWT (on dead *Alnusglutinosa*); IS leg.; • ♂; Vormsi Island; 58°59'26"N, 23°11'51"E; 08.05.–04.06.2012; TWT (on dead *Salixfragilis*); IS leg.; • ♂♂; Vormsi Island; 59°01'01"N, 23°12'18"E; 06.07.–03.08.2012, 03.08.–01.09.2012; TWT (on *Pinussylvestris*); IS leg. **Pärnumaa** • ♂; Matsalu NP; 58°42'41"N, 23°41'19"E; 29.05.–17.06.2009; TWT (on *Populustremula*); IS leg.; • ♂♂; Kihnu Island; 58°08'07"N, 23°58'20"E; 06.08.–13.09.2011, 09.08.–08.09.2012; TWT (on *Pinussylvestris* with *Phellinuspini*); IS leg.; • ♂; Kihnu Island; 58°08'16"N, 23°58'24"E; 06.08.–13.09.2011; TWT (on dead *Pinussylvestris*); IS leg.; • ♂; Kihnu Island; 58°08'28"N, 23°58'47"E; 11.06.–12.07.2012; TWT (on *Pinussylvestris*); IS leg.; • ♂; Luitemaa NR near Võiste; 58°12'21"N, 24°29'36"E; 23.06.–25.07.2017; TWT (on burned *Pinussylvestris*); IS leg.; • ♂; Laulaste NR; 57°59'41"N, 24°32'16"E; 17.06.–03.07.2013; TWT (on dead *Piceaabies* with *Fomitopsispinicola*); IS leg.; • ♂♂; Laulaste NR; 57°59'23"N, 24°33'15"E; 17.06.–03.07.2013, 03.–18.07.2013, 02.–18.08.2013; TWT (on dead *Pinussylvestris*); IS leg.; • ♂♂; Laulaste NR; 57°59'35"N, 24°33'35"E; 04.–17.06.2013, 03.–18.07.2013, 02.–18.08.2013; TWT (on *Betulapendula*); IS leg.; • ♂; Kalita NR, 58°04'15"N, 24°51'37"E; 25.07.–24.08.2017; TWT (on *Populustremula*); IS leg. **Harjumaa** • ♂; Põhja-Kõrvemaa NR; 59°25'30"N, 25°39'11"E; 16.06.–01.07.2015; TWT (on dead *Pinussylvestris*); IS leg. **Lääne–Virumaa** • ♂♂; near Lasila, forest patch No 104; 59°16'43"N, 26°17'07"E; 30.05.–02.07.2015, 12.–30.05.2015, 02.07.–02.08.2015; MT; OK leg.; • ♂♂; near Lasila, forest patch No 24; 59°16'27"N, 26°11'44"E; 30.05.–02.07.2015, 02.–24.08.2015; MT; OK leg.; • ♂♂; near Lasila, forest patch No 31; 59°15'48"N, 26°14'17"E; 12.–30.05.2015, 02.07.–02.08.2015, 02.–24.08.2015, 24.08.–20.09.2015; MT; OK leg.; • ♂♂; near Lasila, forest patch No 50; 59°16'25"N, 26°14'02"E; 02.08.–24.08.2015, 24.08.–20.09.2015; MT; OK leg.; • ♂; near Lasila, forest patch No 52; 59°16'20"N, 26°13'52"E; 26.09.–27.10.2016; MT; OK leg.; • ♂♂; near Lasila, forest patch No 6; 59°15'41"N, 26°12'32"E; 12.–30.05.2015, 29.08.–26.09.2016; MT; OK leg.; • ♂; near Lasila, forest patch No 7; 59°15'33"N, 26°12'41"E; 02.07.–02.08.2015; MT; OK leg.; • ♂; near Lasila, forest patch No 75; 59°16'49"N, 26°13'05"E; 12.–30.05.2015; MT; OK leg.; • ♂; near Lasila, forest patch No 79; 59°16'37"N, 26°13'22"E; 02.07.–02.08.2015; MT; OK leg. **Ida-Virumaa** • ♂; Muraka NR; 59°10'50"N, 27°09'44"E; 27.07.–29.08.2017; TWT (on dead *Populustremula*); IS leg.; • ♂; Kurtna PL; 59°18'22"N, 27°33'53"E; 29.07.–14.08.2015; TWT (on dead *Pinussylvestris*); IS leg.; • ♂; Agusalu NR; 59°02'16"N, 27°39'35"E; 14.08.–01.09.2015; TWT (on dead *Pinussylvestris*); IS leg.; • ♂♂; Agusalu NR; 59°02'17"N, 27°39'40"E; 15.–29.07.2015, 29.07.–14.08.2015; TWT (on dead *Pinussylvestris*); IS leg.; • ♂; Agusalu NR; 59°02'15"N, 27°39'42"E; 15.–30.06.2015; TWT (on dead *Betulapendula*); IS leg. **Jõgevamaa** • ♂♂; Kääpa NR; 58°38'58"N, 26°51'12"E; 15.–30.06.2013, 31.07.–15.08.2013; TWT (on dead *Pinussylvestris*); IS leg. **Tartumaa** • ♂♂; Välgi NR; 58°33'48"N, 26°52'38"E; 14.05.–30.05.2015, 29.07.–14.08.2015; TWT (on *Populustremula*); IS leg.; • ♂; Padakõrve NR; 58°36'08"N, 26°58'09"E; 31.07.–15.08.2013; TWT (on *Piceaabies*); IS leg.; • ♂; Padakõrve NR; 58°35'38"N, 26°57'52"E; 15.08.–04.09.2013; TWT (on dead *Pinussylvestris*); IS leg.; • ♂♂; Padakõrve NR; 58°35'07"N, 27°01'09"E; 14.05–30.05.2015, 14.08–01.09.2015; TWT (on dead *Pinussylvestris*); IS leg.; • ♂♂; Vorbuse, Lokaatorite tee; 58°25'27"N, 26°35'47"E; 14.05.–29.05.2023, 25.06.–14.07.2023, 02.08–16.08.2023, 16.08.–30.08.2023, 08.–16.09.2023, 18.09.–01.10.2023, 01.10.–15.10.2023, 15.10.–29.10.2023; MT; OK leg. **Põlvamaa** • ♂; Ihamaru NR; 58°06'09"N, 26°55'59"E; 27.04.–13.05.2015; TWT (on dead *Betulapendula* with *Fomesfomentarius*); IS leg. **Valgamaa** • ♂; Soontaga; 58°00'04"N, 26°05'11"E; 05.06.–06.07.2015; TWT (on dead *Betulapendula* with *Fomesfomentarius*); IS leg.; • ♂; Koiva wooded meadow; 57°41'26"N, 26°11'06"E; 17.08.–01.09.2013; TWT (on *Quercusrobur*); IS leg.; • ♂; Koivakonnu; 57°35'27"N, 26°19'40"E; 14.05.–03.06.2013; TWT (on dead *Betulapendula* with *Fomesfomentarius*); IS leg.; • ♂♂; Koivakonnu; 57°35'28"N, 26°19'21"E; 16.06.–02.07.2013, 01.–17.08.2013; TWT (on dead *Quercusrobur*); IS leg.

**Figure 3.**
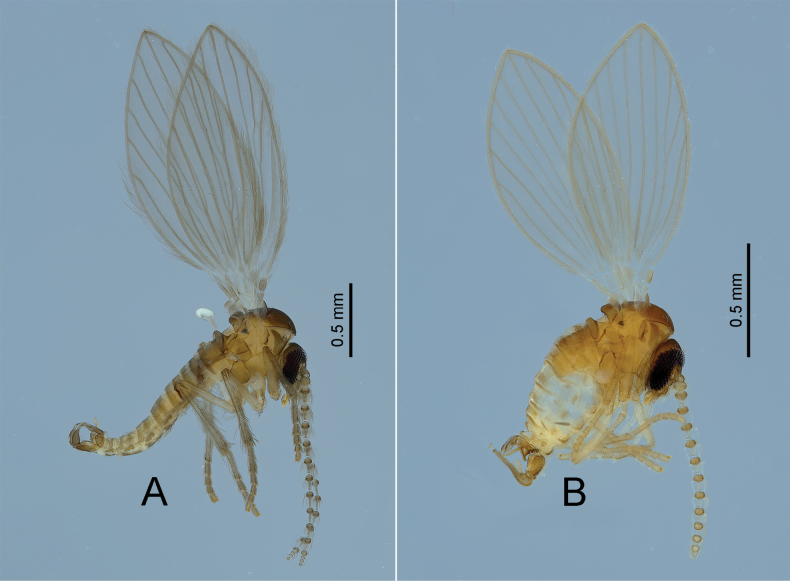
Lateral habitus of Estonian moth flies: the tribe Psychodini**A***Psychodochacinerea* (Banks, 1894) **B***Psychodulaminuta* (Banks, 1894).

###### Comments.

Cosmopolitan species (Ježek and Yağci 2005; Afzan and Belqat 2016; Ježek et al. 2020, 2021a, 2023b).

##### ﻿*32. Psychodocha gemina

Taxon classificationAnimaliaDipteraPsychodidae

(Eaton, 1904)

799526DF-7CA5-54E6-ACAD-4311F3900AF9

###### Material examined.

**Saaremaa** • ♂; Ruhnu Island, near Korsi farm; 57°48'28"N, 23°14'32"E; 03.08.–11.09.2011; TWT (on dead *Fraxinusexcelsior*); IS leg. **Läänemaa** • ♂; Vormsi Island, Suuremõisa Park; 58°59'30"N, 23°11'34"E; 06.07.–19.07.2011; TWT (on *Quercusrobur*); IS leg.; • ♂; Puise; 58°47'45"N, 23°31'22"E; 11.07.–14.08.2009; TWT (on old *Betulapendula*); IS leg.; • ♂; Puise; 58°47'41"N, 23°31'28"E; 17.06.–11.07.2009; TWT (on dead *Alnusincana* with *Fomitopsispinicola*); IS leg. **Pärnumaa** • ♂; Riimaru; 58°16'18"N, 25°11'02"E; 13.07.–03.08.2005; TWT (on dead *Betulapendula* with *Fomesfomentarius*); IS leg. **Lääne-Virumaa** • ♂; Lahemaa NP, near Laukasoo; 59°28'49"N, 25°54'24"E; 23.05.–23.06.2017; TWT (on dead *Populustremula*); IS leg.; • ♂♂; near Lasila, forest patch No 104; 59°16'43"N, 26°17'07"E; 20.09.–21.10.2015, 28.07.–29.08.2016; MT, OK leg.; • ♂♂; near Lasila, forest patch No 24; 59°16'27"N, 26°11'44"E; 05.–31.05.2016, 31.05.–28.06.2016, 28.07.–29.08.2016; MT; OK leg.; • ♂; near Lasila, forest patch No 31; 59°15'48"N, 26°14'17"E; 31.05.–28.06.2016; MT; OK leg.; • ♂♂; near Lasila, forest patch No 50; 59°16'25"N, 26°14'02"E; 05.–31.05.2016, 31.05.–28.06.2016, 28.06.–28.07.2016, 29.08.–26.09.2016; MT; OK leg.; • ♂; near Lasila, forest patch No 52; 59°16'20"N, 26°13'52"E; 29.08.–26.09.2016; MT; OK leg.; • ♂; near Lasila, forest patch No 6; 59°15'41"N, 26°12'32"E; 28.07.–29.08.2016; MT; OK leg.; • ♂; near Lasila, forest patch No 7; 59°15'33"N, 26°12'42"E; 29.08.–26.09.2016; MT; OK leg.; • ♂; near Lasila, forest patch No 75; 59°16'49"N, 26°13'05"E; 05.–31.05.2016, 28.07.–29.08.2016; MT; OK leg.; • ♂♂; near Lasila, forest patch No 79; 59°16'37"N, 26°13'22"E; 05.–31.05.2016, 28.06.–28.07.2016, 28.07.–29.08.2016, 29.08.–26.09.2016; MT; OK leg.; • ♂; Tudusoo NR; 59°04'47"N, 26°45'38"E; 27.07.–29.08.2017; TWT; IS leg. **Ida-Virumaa** • ♂; Muraka NR; 59°05'22"N, 27°09'18"E; 30.04.–14.05.2015; TWT (on dead *Piceaabies*); IS leg. **Jõgevamaa** • ♂; Kääpa NR; 58°38'58"N, 26°51'12"E; 15.07.–31.07.2013; TWT (on dead *Pinussylvestris*); IS leg. **Tartumaa** • ♂; Padakõrve NR; 58°35'07"N, 27°01'09"E; 15.06.–30.06.2015; TWT (on dead *Pinussylvestris*); IS leg.; • ♂; Rootsiküla; 58°37'14"N, 27°10'47"E; 10.05.–06.06.2009; TWT (on dead *Salixcaprea*); IS leg.; • ♂♂; Vorbuse, Lokaatorite tee; 58°25'27"N, 26°35'47"E; 30.04.–14.05.2023, 14.05.–29.05.2023, 10.06.–25.06.2023; MT; OK leg. **Põlvamaa** • ♂; Ihamaru NR; 58°06'11"N, 26°55'55"E; 13.–31.08.2015; TWT (on dead *Betulapendula*); IS leg. **Valgamaa** • ♂♂; Soontaga; 58°00'03"N, 26°05'31"E; 11.05.–05.06.2015, 05.06.–06.07.2015, 06.07.–03.08.2015; TWT (on *Acerplatanoides*); IS leg.; • ♂; Soontaga NR; 58°01'19"N, 26°04'01"E; 28.07.–31.08.2015; TWT (on old *Quercusrobur*); IS leg.; • ♂; Koiva wooded meadow; 57°41'26"N, 26°11'06"E; 14.05.–03.06.2013; TWT (on *Quercusrobur*); IS leg.; • ♂; Koiva wooded meadow; 57°41'21"N, 26°11'08"E; 16.06.–02.07.2013; TWT (on dead *Quercusrobur*); IS leg.; • ♂; Koivakonnu; 57°35'28"N, 26°19'21"E; 17.08.–01.09.2013; TWT (on dead *Quercusrobur*); IS leg.

###### Comments.

European and Transcaucasian species (Ježek et al. 2019, 2020, 2021a).

##### ﻿*33. Psychodula minuta

Taxon classificationAnimaliaDipteraPsychodidae

(Banks, 1894)

FED0210C-7F6A-5820-BD5E-BFDD6EA2772E

Fig. 3B

###### Material examined.

**Pärnumaa** • ♂; Kihnu Island; 58°07'49"N, 24°00'15"E; 22.05.–11.06.2012; TWT (on *Quercusrobur*); IS leg.; • ♂; Laulaste NR; 57°59'23"N, 24°33'15"E; 18.–31.08.2013; TWT (on dead *Pinussylvestris*); IS leg.; • ♂; Laulaste NR; 57°59'49"N, 24°33'30"E; 17.06.–03.07.2013; TWT (on *Pinussylvestris*); IS leg.; • ♂; Laulaste NR; 57°59'35"N, 24°33'35"E; 18.07.–02.08.2013; TWT (on *Betulapendula*); IS leg. **Lääne-Virumaa** • ♂; near Lasila, forest patch No 104; 59°16'43"N, 26°17'07"E; 29.08.–26.09.2016; MT; OK leg.; • ♂; near Lasila, forest patch No 31; 59°15'48"N, 26°14'17"E; 20.09.–21.10.2015; MT; OK leg.; • ♂; near Lasila, forest patch No 50; 59°16'25"N, 26°14'02"E; 28.07.–29.08.2016; MT; OK leg.; • ♂♂; near Lasila, forest patch No 79; 59°16'37"N, 26°13'22"E; 12.–30.05.2015, 20.09.–21.10.2015; MT; OK leg. **Ida-Virumaa** • ♂; Agusalu NR; 59°02'16"N, 27°39'35"E; 30.05.–15.06.2015; TWT (on dead *Pinussylvestris*); IS leg.; • ♂; Agusalu NR; 59°02'17"N, 27°39'40"E; 29.07.–14.08.2015; TWT (on dead *Pinussylvestris*); IS leg. **Jõgevamaa** • ♂; Kääpa NR; 58°38'58"N, 26°51'12"E; 15.–31.07.2013; TWT (on dead *Pinussylvestris*); IS leg.; • ♂; Kääpa NR; 58°39'01"N, 26°51'09"E; 15.08.–04.09.2013; TWT (on *Pinussylvestris*); IS leg. **Valgamaa** • ♂; Soontaga; 58°00'03"N, 26°05'31"E; 05.06.–06.07.2015; TWT (on *Acerplatanoides*); IS leg.

###### Comments.

Holarctic species (Ježek et al. 2019, 2020, 2023b).

##### ﻿*34. Tinearia alternata

Taxon classificationAnimaliaDipteraPsychodidae

(Say, 1824)

2ABDC388-718C-5254-95FE-B62013519A1B

###### Material examined.

**Saaremaa** • ♀; Ruhnu Island; 57°48'20"N, 23°14'33"E; 07.08.–05.09.2012; TWT (on dead *Alnusglutinosa*); IS leg. **Läänemaa** • ♀; Vormsi Island; 58°58'33"N, 23°12'10"E; 03.08.–01.09.2012; TWT (on dead *Betulapendula*); IS leg. **Pärnumaa** • ♀; Kihnu Island, Lemsi; 58°07'49"N, 24°00'15"E; 06.08.–13.09.2011; TWT (on old *Quercusrobur*); IS leg.; • ♀; Kihnu Island; 58°08'28"N, 23°58'47"E; 12.07.–09.08.2012; TWT (on *Pinussylvestris*); IS leg.; • ♂♀; Riimaru; 58°16'18"N, 25°11'02"E; 03.–17.08.2005, 31.07.–02.09.2006; TWT (on *Populustremula*); IS leg. **Tartumaa** • ♂; Tartu, Aardla 124; 58°21'10"N, 26°41'08"E; 13.05.2016; hand picked; T. Kesküla leg.

###### Comments.

Cosmopolitan species (Ježek et al. 2019, 2020, 2023b).

##### ﻿*35. Ypsydocha setigera

Taxon classificationAnimaliaDipteraPsychodidae

(Tonnoir, 1922)

D5D2F659-8152-59E1-B934-433E61A50FE0

###### Material examined.

**Lääne-Virumaa** • ♀; near Lasila, forest patch No 50; 59°16'25"N, 26°14'02"E; 28.07.–29.08.2016; MT; OK leg.; INS 33821.

###### Comments.

A very common Holarctic species (Ježek and Omelková 2012).

#### ﻿Pericomaini

##### ﻿*36. Clytocerus (Boreoclytocerus) longicorniculatus

Taxon classificationAnimaliaDipteraPsychodidae

Krek, 1987

95C60BE5-8FCF-5A5A-A4B7-6464CBAE18BD

###### Material examined.

**Lääne-Virumaa** • ♂; near Lasila, forest patch No 79; 59°16'37"N, 26°13'22"E; 12.–30.05.2015; MT; OK leg.

###### Comments.

A species known only from Europe (Ježek et al. 2020). Important species for nature conservation, assessed as nationally scarce in the Czech Republic.

##### ﻿37. Clytocerus (Boreoclytocerus) ocellaris

Taxon classificationAnimaliaDipteraPsychodidae

(Meigen, 1804)

DC15AAF5-7BF3-5F55-9BC9-3AC48F631746

Fig. 4B

###### Published record.

Salmela and Piirainen (2005): 304.

###### Material examined.

**Saaremaa** • ♂♂; Ruhnu Island, Holma; 57°48'02"N, 23°13'42"E; 29.06.–02.08.2011, 07.08.–05.09.2012; TWT (on old *Ulmuslaevis* with *Polyporussquamosus*); IS leg.; • ♂; Ruhnu Island, near Korsi farm; 57°48'28"N, 23°14'32"E; 03.08.–11.09.2011; TWT (on dead *Fraxinusexcelsior*); IS leg.; • ♂; Ruhnu Island; 57°48'15"N, 23°14'32"E; 07.08.–05.09.2012; TWT (on *Acerplatanoides*); IS leg.; • ♂; Ruhnu Island; 57°48'24"N, 23°15'16"E; 07.08.–05.09.2012; TWT (on dead *Salixcaprea*); IS leg. **Läänemaa** • ♂; Vormsi Island, Suuremõisa park; 58°59'30"N, 23°11'34"E; 06.07.–03.08.2012; TWT (on *Quercusrobur*); IS leg.; • ♂; Vormsi Island; 59°01'01"N, 23°12'18"E; 03.08.–01.09.2012; TWT (on *Pinussylvestris*); IS leg. **Pärnumaa** • ♂; Kihnu Island; 58°06'59"N, 23°59'38"E; 12.07.–09.08.2012; TWT (on old *Quercusrobur*); IS leg.; • ♂; Kihnu Island; 58°07'49"N, 24°00'15"E; 22.05.–11.06.2012; TWT (on old *Quercusrobur*); IS leg.; • ♂♂; Matsalu NP; 58°42'41"N, 23°41'19"E; 29.05.–17.06.2009, 11.07.–14.08.2009; TWT (on *Populustremula*); IS leg.; • ♂; Matsalu NP; 58°42'52"N, 23°41'23"E; 11.07.–14.08.2009; TWT (on dead *Populustremula*); IS leg.; • ♂; Puhtu; 58°33'31"N, 23°33'08"E; 18.06.–10.07.2009; TWT (on dead *Piceaabies*); IS leg.; • ♂; Sookuninga NR; 58°00'08"N, 24°52'38"E; 21.05.–22.06.2017; TWT (on dead *Populustremula*); IS leg. **Harjumaa** • ♂; Mähuste near Koitjärve; 59°24'55"N, 25°36'45"E; 30.07.–15.08.2015; TWT (on dead *Betulapendula*); IS leg. **Lääne-Virumaa** • ♂♂; near Lasila, forest patch No 24; 59°16'27"N, 26°11'44"E; 24.08.–20.09.2015, 05.–31.05.2016, 28.07.–29.08.2016, 29.08.–26.09.2016, 26.09.–27.10.2016; MT; OK leg.; • ♂♂; near Lasila, forest patch No 31; 59°15'48"N, 26°14'17"E; 12.–30.05.2015, 02.07.–02.08.2015, 24.08.–20.09.2015, 28.07.–29.08.2016, 26.09.–27.10.2016; MT; OK leg.; • ♂♂; near Lasila, forest patch No 50; 59°16'25"N, 26°14'02"E; 12.05.–30.05.2015, 29.08.–26.09.2016; MT; OK leg.; • ♂♂; near Lasila, forest patch No 52; 59°16'20"N, 26°13'52"E; 28.07.–29.08.2016, 29.08.–26.09.2016; MT; OK leg.; • ♂♂; near Lasila, forest patch No 6; 59°15'41"N, 26°12'32"E; 28.07.–29.08.2016, 29.08.–26.09.2016; MT; OK leg.; • ♂♂; near Lasila, forest patch No 75; 59°16'49"N, 26°13'05"E; 12.–30.05.2015, 20.09.–21.10.2015, 05.–31.05.2016, 28.07.–29.08.2016; MT; OK leg.; • ♂♂; near Lasila, forest patch No 79; 59°16'37"N, 26°13'22"E; 02.–24.08.2015, 24.08.–20.09.2015, 05.–31.05.2016, 31.05.–28.06.2016, 28.06.–28.07.2016; MT; OK leg.; • ♂; Suigu NR; 59°09'01"N, 26°49'08"E; 26.07.–29.08.2017; TWT (on *Populustremula*); IS leg.; • ♂; Suigu NR; 59°09'11"N, 26°49'10"E; 26.07.–29.08.2017; TWT (on dead *Populustremula*); IS leg.; INS 33808; • ♂; Tudusoo NR; 59°04'47"N, 26°45'38"E; 27.07.–29.08.2017; TWT (on *Populustremula*); IS leg.; • ♂; Tudusoo NR; 59°04'53"N, 26°46'15"E; 26.07.–29.08.2017; TWT (on *Populustremula*); IS leg. **Ida-Virumaa** • ♂; Muraka NR; 59°10'50"N, 27°09'44"E; 25.05.–26.06.2017; TWT (on *Populustremula*); IS leg. **Tartumaa** • ♂; Rootsiküla; 58°37'14"N, 27°10'47"E; 15.07.–02.08.2009; TWT (on dead *Salixcaprea*); IS leg. **Valgamaa** • ♂♂; Soontaga; 58°00'04"N, 26°05'15"E; 05.06.–06.07.2015, 03.–31.08.2015; TWT (on dead *Betulapendula*); IS leg.; • ♂♂; Soontaga; 58°00'04"N, 26°05'11"E; 07.05.–05.06.2015, 05.06–06.07.2015, 03.–31.08.2015; TWT (on dead *Betulapendula* with *Fomesfomentarius*); IS leg.; • ♂♂; Soontaga; 58°00'01"N, 26°05'10"E; 19.05.–05.06.2015, 06.07–03.08.2015; TWT (on dead *Populustremula*); IS leg.; • ♂♂; Koiva wooded meadow; 57°41'21"N, 26°11'12"E; 02.–17.07.2013, 17.08.–01.09.2013; TWT (on dead *Populustremula*); IS leg.; • ♂; Koiva wooded meadow; 57°41'19"N, 26°10'59"E; 14.05.–03.06.2013; TWT (on dead *Quercusrobur*); IS leg.; • ♂; Koivakonnu; 57°35'27"N, 26°19'40"E; 14.05.–03.06.2013; TWT (on dead *Betulapendula* with *Fomesfomentarius*); IS leg.

**Figure 4.**
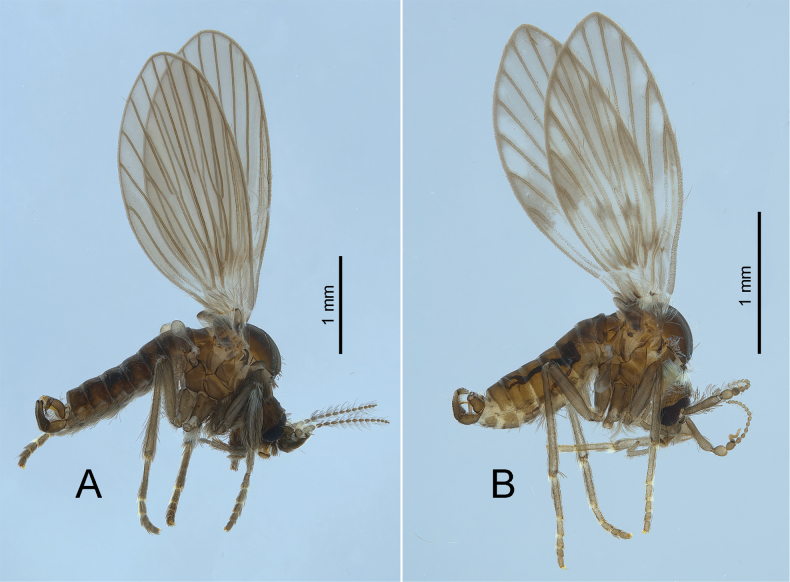
Lateral habitus of Estonian moth flies: the tribe Pericomaini**A***Pneumianubila* (Meigen, 1818) **B**Clytocerus (Boreoclytocerus) ocellaris (Meigen, 1804).

###### Comments.

European species (Ježek et al. 2019, 2020; Morelli and Biscaccianti 2021).

##### ﻿*38. Clytocerus (Boreoclytocerus) splendidus

Taxon classificationAnimaliaDipteraPsychodidae

Ježek & Hájek, 2007

FFAFB684-9CA4-5518-92F6-002CA6E9BF6D

###### Material examined.

**Saaremaa** • ♂; Ruhnu Island; 57°48'01"N, 23°14'38"E; 07.08.–05.09.2012; TWT (on *Acerplatanoides*); IS leg.; • ♂; Ruhnu Island; 57°48'06"N, 23°13'58"E; 07.08.–05.09.2012; TWT (on *Sorbusintermedia* with *Laetiporussulphureus*); IS leg.; • ♂; Ruhnu Island; 57°48'09"N, 23°14'25"E; 07.08.–05.09.2012; TWT (on *Fraxinusexcelsior*); IS leg.; • ♂; Ruhnu Island; 57°48'10"N, 23°14'24"E; 07.08.–05.09.2012; TWT (on *Fraxinusexcelsior*); IS leg.; • ♂; Ruhnu Island, Holma; 57°48'02"N, 23°13'42"E; 07.06.–10.07.2012; TWT (on old *Ulmuslaevis*); IS leg. **Läänemaa** • ♂♂; Vormsi Island, 58°59'26"N, 23°11'51"E; 08.05.–04.06.2012, 06.07.–03.08.2012; TWT (on *Salixfragilis*); IS leg.; • ♂; Vormsi Island; 59°01'20"N, 23°08'13"E; 09.05.–04.06.2012; TWT (on dead *Alnusglutinosa*); IS leg. **Pärnumaa** • ♂; Kihnu Island; 58°08'07"N, 23°58'20"E; 09.08.–08.09.2012; TWT (on *Pinussylvestris* with *Phellinuspini*); IS leg. **Lääne-Virumaa** • ♂; near Lasila, forest patch No 24; 59°16'27"N, 26°11'44"E; 12.–30.05.2015; MT; OK leg.; • ♂♂; near Lasila, forest patch No 31; 59°15'48"N, 26°14'17"E; 12.–30.05.2015, 02.07.–02.08.2015, 02.–24.08.2015, 20.09.–21.10.2015; MT; OK leg.; • ♂; near Lasila, forest patch No 50; 59°16'25"N, 26°14'02"E; 24.08.–20.09.2015; MT; OK leg.; • ♂; near Lasila, forest patch No 75; 59°16'49"N, 26°13'05"E; 02.–24.08.2015; MT; OK leg. **Tartumaa** • ♂; Rootsiküla; 58°37'14"N, 27°10'47"E; 15.07.–02.08.2009; TWT (on dead *Salixcaprea*); IS leg.; INS 33809. **Valgamaa** • ♂; Koiva wooded meadow; 57°41'21"N, 26°11'12"E; 17.07.–01.08.2013; TWT (on dead *Populustremula*); IS leg.

###### Comments.

Not common European species (Ježek et al. 2018, 2019; Kroča and Ježek 2019). Important species for nature conservation, assessed as nationally scarce in the Czech Republic.

##### ﻿39. Clytocerus (Boreoclytocerus) tetracorniculatus

Taxon classificationAnimaliaDipteraPsychodidae

Wagner, 1977

84AA82B7-1025-56E5-8C08-079AB60B3671

###### Published record.

Salmela and Piirainen (2005): 304.

###### Comments.

Not common European species (Kroča and Ježek 2019). Important species for nature conservation, assessed as critically endangered in the Czech Republic.

##### ﻿*40. Pericoma albomaculata

Taxon classificationAnimaliaDipteraPsychodidae

Wahlgren, 1904

CB44FEBE-F450-50DB-9DA4-CC135AEB75E9

###### Note.

Junior synonymous name: *Pneumiarivularis* (Berdén, 1954), see Kvifte et al. (2020).

###### Material examined.

**Saaremaa** • ♂; Ruhnu Island, near Korsi farm; 57°48'28"N, 23°14'32"E; 26.05.–28.06.2011; TWT (on dead *Fraxinusexcelsior*); IS leg.; • ♂; Ruhnu Island; 57°47'59"N, 23°14'34"E; 07.06.–10.07.2012; TWT (on dead *Fraxinusexcelsior*); IS leg.; • ♂; Ruhnu Island; 57°48'01"N, 23°14'38"E; 07.06.–10.07.2012; TWT (on *Acerplatanoides*); IS leg.; • ♂; Ruhnu Island; 57°48'15"N, 23°14'32"E; 07.06.–10.07.2012; TWT (on *Acerplatanoides*); IS leg. **Läänemaa** • ♂; Vormsi Island, Suuremõisa Park; 58°59'30"N, 23°11'34"E; 06.06.–06.07.2011; TWT (on old *Quercusrobur*); IS leg. **Pärnumaa** • ♂; Matsalu NP, 58°42'41"N, 23°41'19"E; 11.07.–14.08.2009; TWT (on *Populustremula*); IS leg.; • ♂; Kalita NR; 58°04'15"N, 24°51'37"E; 25.07.–24.08.2017; TWT (on *Populustremula*); IS leg. **Lääne-Virumaa** • ♂♂; near Lasila, forest patch No 31; 59°15'48"N, 26°14'17"E; 12.–30.05.2015, 02.07.–02.08.2015; MT; OK leg.; • ♂; near Lasila, forest patch No 50; 59°16'25"N, 26°14'02"E; 12.–30.05.2015; MT; OK leg.; • ♂; near Lasila, forest patch No 79; 59°16'37"N, 26°13'22"E; 30.05.–02.07.2015; MT; OK leg.; • ♂; Tudusoo NR; 59°04'47"N, 26°45'38"E; 28.05.–25.06.2017; TWT; IS leg.; • ♂; Tudusoo NR; 59°04'53"N, 26°46'15"E; 27.05.–25.06.2017; TWT (on *Populustremula*); IS leg.; INS 33781. **Ida-Virumaa** • ♂; Muraka NR, Mädamänniku; 59°07'07"N, 27°13'08"E; 25.05.–26.06.2017; TWT (on dead *Populustremula*); IS leg.; INS 33799.

###### Comments.

We follow the latest formal interpretation of this species by Kvifte et al. (2020). However, we consider the synonymy provided above questionable. As addressing it is beyond the scope of this paper, we postpone a detailed discussion to a future work. A Palaearctic species (Ježek et al. 2019) assessed as endangered in Czech Republic, but found to be common in Finland (Salmela et al. 2007; Salmela 2008).

##### ﻿*41. Pneumia compta

Taxon classificationAnimaliaDipteraPsychodidae

(Eaton, 1893)

DB85B28A-21AD-5FC3-9ECD-B990E45495C7

###### Material examined.

**Lääne-Virumaa** • ♂; near Lasila, forest patch No 79; 59°16'37"N, 26°13'22"E; 30.05.–02.07.2015; MT; OK leg. **Ida–Virumaa** • ♂; Agusalu NR; 59°04'16"N, 27°37'40"E; 14.05.–30.05.2015; TWT (on dead *Betulapendula* with *Fomesfomentarius*); IS leg.; INS 33805.

###### Comments.

A Palaearctic species (Oboňa and Ježek 2014; Ježek et al. 2019; Morelli and Biscaccianti 2022). Important species for nature conservation, assessed as nationally scarce in the Czech Republic.

##### ﻿42. Pneumia mutua

Taxon classificationAnimaliaDipteraPsychodidae

(Eaton, 1893)

12C54A22-CEA7-520E-9C1F-7C31DD2BED02

###### Published record.

Salmela and Piirainen (2005): 304.

###### Comments.

European species (Ježek et al. 2019).

##### ﻿*43. Pneumia nubila

Taxon classificationAnimaliaDipteraPsychodidae

(Meigen, 1818)

F6F4936D-C563-5E6B-8EA4-AB3BD2DF8D9B

Fig. 4A

###### Material examined.

**Saaremaa** • ♂; Ruhnu Island; 57°47'59"N, 23°14'34"E; 26.05.–28.06.2011; TWT (on dead *Fraxinusexcelsior*); IS leg.; • ♂; Ruhnu Island; 57°48'15"N, 23°14'32"E; 07.08.–05.09.2012; TWT (on *Acerplatanoides*); IS leg.; • ♂; Ruhnu Island; 57°48'18"N, 23°14'27"E; 07.08.–05.09.2012; TWT (on *Quercusrobur*); IS leg. **Läänemaa** • ♂; Puise; 58°47'45"N, 23°31'22"E; 17.06.–11.07.2009; TWT (on old *Betulapendula*); IS leg. **Pärnumaa** • ♂; Matsalu NP, 58°42'52"N, 23°41'23"E; 29.05.–17.06.2009; TWT (on dead *Populustremula*); IS leg. **Harjumaa** • ♂; Lahemaa NP, Palanselja; 59°32'44"N, 25°39'31"E; 26.07.–26.08.2017; TWT (on burned *Pinussylvestris*); IS leg.; • ♂; Põhja-Kõrvemaa NR; 59°25'30"N, 25°39'11"E; 15.05.–01.06.2015; TWT (on dead *Pinussylvestris*); IS leg. **Lääne-Virumaa** • ♂; Lahemaa NP, near Laukasoo; 59°28'49"N, 25°54'24"E; 25.07.–26.08.2017; TWT (on dead *Populustremula*); IS leg. **Tartumaa** • ♂; Elva-Vitipalu PL; 58°10'49"N, 26°25'14"E; 27.04.–13.05.2015; TWT (on dead *Piceaabies*); IS leg.

###### Comments.

Common European species (Ježek et al. 2019, 2020, 2021b), known also from Transcaucasia (Ježek et al. 2023a).

##### ﻿*44. Pneumia trivialis

Taxon classificationAnimaliaDipteraPsychodidae

(Eaton, 1893)

BDB8D2BB-4512-5E26-9DF2-E83A6C792896

###### Material examined.

**Saaremaa** • ♂♂; Ruhnu Island; 57°47'59"N, 23°14'34"E; 03.08.–11.09.2011, 07.08.–05.09.2012; TWT (on dead *Fraxinusexcelsior*); IS leg. **Pärnumaa** • ♂; Matsalu NP; 58°42'41"N, 23°41'19"E; 29.05.–17.06.2009; TWT (on *Populustremula*); IS leg. **Valgamaa** • ♂; Soontaga NR; 58°00'23"N, 26°03'19"E; 13.05.–29.05.2015; TWT (on dead *Piceaabies*); IS leg.; • ♂; Soontaga NR; 58°01'19"N, 26°04'01"E; 29.05.–29.06.2015; TWT (on old *Quercusrobur*); IS leg.

###### Comments.

European and Transcaucasian species (Ježek et al. 2019, 2020, 2021a, 2023a).

##### ﻿*45. Tonnoiriella nigricauda

Taxon classificationAnimaliaDipteraPsychodidae

(Tonnoir, 1919)

1EE6B9AB-1DD6-5A13-A6FE-81919CC1BBE2

###### Material examined.

**Pärnumaa** • ♂; Matsalu NP; 58°42'41"N, 23°41'19"E; 29.05.–17.06.2009; TWT (on *Populustremula*); IS leg. **Lääne-Virumaa** • ♂; near Lasila, forest patch No 79; 59°16'37"N, 26°13'22"E; 28.06.–28.07.2016; MT; OK leg.; INS 33791. **Valgamaa** • ♂; Soontaga; 58°00'04"N, 26°05'11"E; 05.06.–06.07.2015; TWT (on dead *Betulapendula* with *Fomesfomentarius*); IS leg.; • ♂; Soontaga; 58°00'04"N, 26°05'15"E; 03.08.–31.08.2015; TWT (on dead *Betulapendula*); IS leg.; • ♂; Soontaga; 58°00'03"N, 26°05'31"E; 06.07.–03.08.2015; TWT (on *Acerplatanoides*); IS leg.

###### Comments.

European species (Kvifte et al. 2011; Wagner 2018; Ježek et al. 2019). Important species for nature conservation, assessed as critically endangered in the Czech Republic.

## ﻿Discussion

Given the limited knowledge regarding moth fly diversity in the Baltic region, the discovery of 30 new country records in Estonia comes as no surprise. The majority of the recorded species (31 of 45; original data combined with Salmela and Piirainen (2005)) have been collected only from 1–3 localities (including 16 from a single locality), emphasizing the urgent need for further study. Conversely, seven recorded species are more widespread, having been collected from more than ten localities. Currently, a total of 71 species are documented in the Baltic countries (Table 1), while 63 species are known from Finland (including two of them marked as questionable; Salmela et al. 2014). The only species known from Latvia, Clytocerus (B.) ocellaris, is widely distributed in Europe as well as in the Nordic-Baltic region (Ježek and Goutner 1995).

**Table 1.** An updated systematic list of the psychodids of the Baltic Countries. Lithuanian data are derived from Pakalniškis et al. (2006) and Latvian data from Salmela and Vartija (2007).

Species	Estonia	Latvia	Lithuania
*Sycoraxsilacea* Haliday in Curtis, 1839	x		
*Trichomyiaurbica* Haliday in Curtis, 1839	x*		
*Oomormiaandrenipes* (Strobl, 1910)	x*		
*Lepimormiahemiboreale* Salmela & Piirainen, 2005	x		
*Promormiaeatoni* (Tonnoir, 1940)	x*		
*Lepiseodinarothschildi* (Eaton, 1912)	x*		x
*Lepiseodinatristis* (Meigen, 1830)			x
*Panimerusalbifacies* (Tonnoir, 1919)	x		
*Panimerusgoetghebueri* (Tonnoir, 1919)			x
*Panimerusnotabilis* (Eaton, 1893)	x*		
*Parajungiellaconsors* (Eaton, 1893)	x		x
*Parajungiellalongicornis* (Tonnoir, 1919)	x*		
*Parajungiellapseudolongicornis* (Wagner, 1975)	x		
*Parajungiellaserbica* (Krek, 1985)	x*		
Paramormia (Paramormia) polyascoidea (Krek, 1971)	x		
Paramormia (Phyllotelmatoscopus) decipiens (Eaton, 1893)			x
*Peripsychodaauriculata* (Haliday in Curtis, 1839)	x		
*Peripsychodafusca* (Macquart, 1826)			x
*Psycmeraintegella* (Jung, 1956)			x
*Seodabritteni* (Tonnoir, 1940)			x
*Seodacarthusiana* (Vaillant, 1972)	x		
*Seodagressica* (Vaillant, 1972)	x*		
*Seodalabeculosa* (Eaton, 1893)	x*		x
*Seodamorula* (Eaton, 1893)			x
*Feuerborniellaobscura* (Tonnoir, 1919)	x		
Philosepedon (Philosepedon) austriacum Vaillant, 1974	x*		
Philosepedon (Philosepedon) dumosum Omelková & Ježek, 2012	x*		
Philosepedon (Philosepedon) humerale (Meigen, 1818)	x		x
Philosepedon (Philothreticus) soljani Krek, 1971	x*		
Philosepedon (Trichosepedon) balkanicum Krek, 1970	x*		
*Threticuslucifugus* (Walker, 1856)			x
*Trichopsychodahirtella* (Tonnoir, 1919)	x*		
*Apsychapusilla* (Tonnoir, 1922)			x
*Chodopsychabuxtoni* (Withers, 1988)	x*		
*Chodopsychalobata* (Tonnoir, 1940)	x*		x
*Copropsychodabrevicornis* (Tonnoir, 1940)			x
*Logimaalbipennis* (Zetterstedt, 1850)	x		x
*Logimasatchelli* (Quate, 1955)	x*		
*Logimasigma* (Kincaid, 1899)	x*		
*Psychagrisescens* (Tonnoir, 1922)			x
*Psychodaphalaenoides* (Linnaeus, 1758)	x		x
*Psychodochacinerea* (Banks, 1894)	x		x
*Psychodochagemina* (Eaton, 1904)	x*		x
*Psychodulaminuta* (Banks, 1894)	x*		x
*Tineariaalternata* (Say, 1824)	x*		x
*Ypsydochasetigera* (Tonnoir, 1922)	x*		x
*Berdeniellamanicata* (Tonnoir, 1920)			x
Clytocerus (Boreoclytocerus) longicorniculatus Krek, 1987	x*		
Clytocerus (Boreoclytocerus) ocellaris (Meigen, 1804)	x	x	x
Clytocerus (Boreoclytocerus) rivosus (Tonnoir, 1919)			x
Clytocerus (Boreoclytocerus) splendidus Ježek & Hájek, 2007	x*		
Clytocerus (Boreoclytocerus) tetracorniculatus Wagner, 1977	x		
*Parabazarellasubneglecta* (Tonnoir, 1922)			x
Pericoma (Pachypericoma) blandula Eaton, 1893			x
Pericoma (Pericoma) albomaculata Wahlgren, 1904	x*		
Pericoma (Pericoma) diversa Tonnoir, 1919			x
Pericoma (Pericoma) trifasciata (Meigen, 1804)			x
*Pneumiacanescens* (Meigen, 1804)			x
*Pneumiacubitospinosa* (Jung, 1954)			x
*Pneumiacompta* (Eaton, 1893)	x*		
*Pneumiaextricata* (Eaton, 1893)			x
*Pneumiamutua* (Eaton, 1893)	x		x
*Pneumianubila* (Meigen, 1818)	x*		x
*Pneumiapalustris* (Meigen, 1804)			x
*Pneumiapilularia* (Tonnoir, 1940)			x
*Pneumiatrivialis* (Eaton, 1893)	x*		
*Tonnoiriellanigricauda* (Tonnoir, 1919)	x*		
*Tonnoiriellapulchra* (Eaton, 1893)			x
*Ulomyiaannulataannulata* (Tonnoir, 1919)			x
*Ulomyiacognata* (Eaton, 1893)			x
*Ulomyiafuliginosa* (Meigen, 1804)			x

* indicates a new country-record.

**Figure 5.**
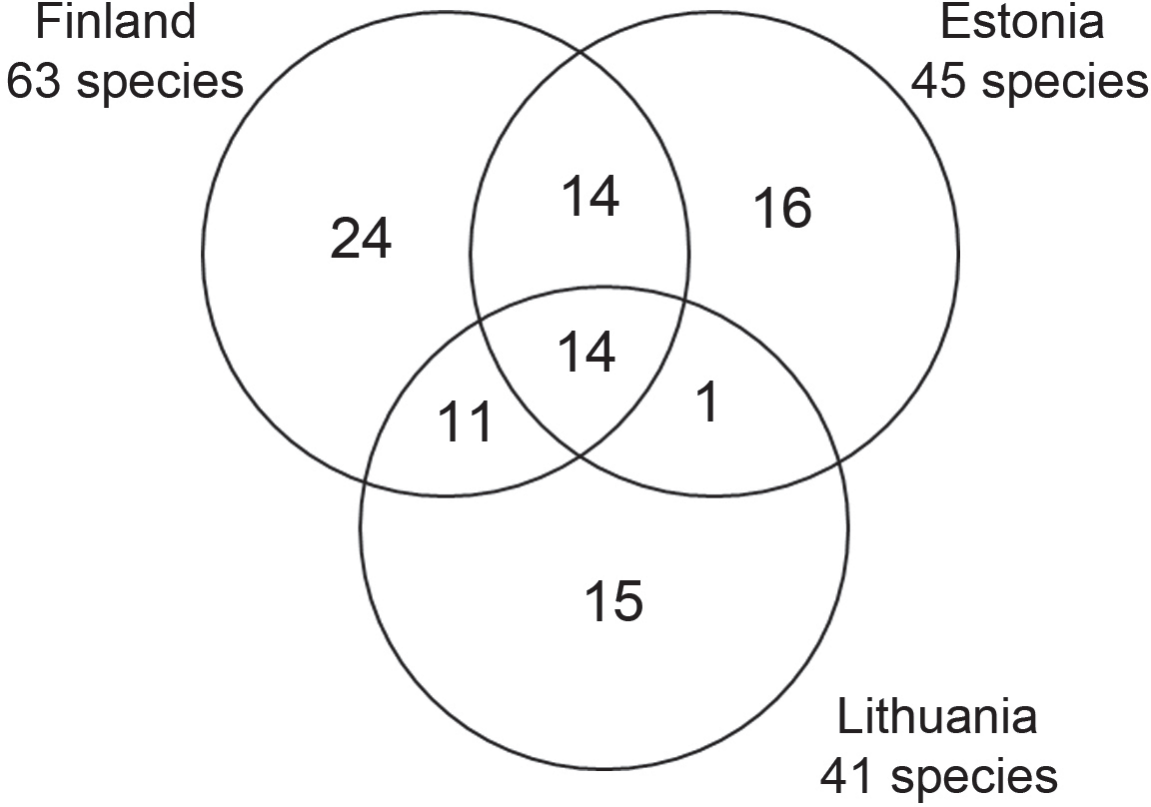
Comparison of the species diversity of moth flies in Estonia, Lithuania, and Finland. The numbers represent recorded species.

When comparing the species lists of Lithuania, Estonia, and Finland, it is noteworthy that all countries have a remarkable proportion of unique species (Fig. 5). Considering this, it is highly likely that the number of species documented in Estonia will continue to increase. Eleven species, which are known to inhabit both Lithuania and Finland (as depicted in Fig. 3), could potentially also be found in Estonia, as there appear to be no apparent limits to their distribution. Interestingly, the genus *Ulomyia* Walker, 1856, which is known from three species in Lithuania, is absent from records in Estonia. It is possible that conducting more intensive research on the surrounding water ecosystems, including both flowing and standing water, could help fill this gap.

Sixteen Estonian species have a conservation status in Central Europe, particularly in the Czech Republic, including six species (viz. *T.urbica*, *O.andrenipes*, *P.pseudolongicornis*, P. *serbica*, C. (B.) tetracorniculatus, *T.nigricauda*) classified as critically endangered, three (viz. *P.eatoni*, *P.albomaculata*, *S.labeculosa*) as endangered, and seven (viz. *L.rothschildi*, P. (P.) soljani, *Ch.buxtoni*, C. (B.) longicorniculatus, C. (B.) splendidus, P. (P.) dumosum, *P.compta*) as nationally scarce. However, one species, *P.albomaculata*, considered endangered in the Czech Republic has been found to be very common in aquatic environments in Finland (Salmela et al. 2007; Salmela 2008), highlighting the importance of sampling across a wide range of habitats. Nevertheless, given that a significant proportion of the studied material was collected from protected areas in Estonia, the knowledge regarding the potential conservation status of these species can be applied in the country’s nature protection management.

## Supplementary Material

XML Treatment for Sycorax silacea

XML Treatment for Trichomyia urbica

XML Treatment for Oomormia andrenipes

XML Treatment for Lepimormia hemiboreale

XML Treatment for Promormia eatoni

XML Treatment for Lepiseodina rothschildi

XML Treatment for Panimerus albifacies

XML Treatment for Panimerus notabilis

XML Treatment for Parajungiella consors

XML Treatment for Parajungiella longicornis

XML Treatment for Parajungiella pseudolongicornis

XML Treatment for Parajungiella serbica

XML Treatment for Paramormia (Paramormia) polyascoidea

XML Treatment for Peripsychoda auriculata

XML Treatment for Seoda carthusiana

XML Treatment for Seoda gressica

XML Treatment for Seoda labeculosa

XML Treatment for Feuerborniella obscura

XML Treatment for Philosepedon (Philosepedon) austriacum

XML Treatment for Philosepedon (Philosepedon) dumosum

XML Treatment for Philosepedon (Philosepedon) humerale

XML Treatment for Philosepedon (Philothreticus) soljani

XML Treatment for Philosepedon (Trichosepedon) balkanicum

XML Treatment for Trichopsychoda hirtella

XML Treatment for Chodopsycha buxtoni

XML Treatment for Chodopsycha lobata

XML Treatment for Logima albipennis

XML Treatment for Logima satchelli

XML Treatment for Logima sigma

XML Treatment for Psychoda phalaenoides

XML Treatment for Psychodocha cinerea

XML Treatment for Psychodocha gemina

XML Treatment for Psychodula minuta

XML Treatment for Tinearia alternata

XML Treatment for Ypsydocha setigera

XML Treatment for Clytocerus (Boreoclytocerus) longicorniculatus

XML Treatment for Clytocerus (Boreoclytocerus) ocellaris

XML Treatment for Clytocerus (Boreoclytocerus) splendidus

XML Treatment for Clytocerus (Boreoclytocerus) tetracorniculatus

XML Treatment for Pericoma albomaculata

XML Treatment for Pneumia compta

XML Treatment for Pneumia mutua

XML Treatment for Pneumia nubila

XML Treatment for Pneumia trivialis

XML Treatment for Tonnoiriella nigricauda

## ﻿Additional information

### Author ORCIDs

Jozef Oboňa https://orcid.org/0000-0002-1185-658X

Olavi Kurina https://orcid.org/0000-0002-4858-4629

Jan Ježek https://orcid.org/0000-0001-5993-4287

### Data availability

All of the data that support the findings of this study are available in the main text.
